# Pyrazolo[4,3-*e*]tetrazolo[1,5-*b*][1,2,4]triazine Sulfonamides as an Important Scaffold for Anticancer Drug Discovery—In Vitro and In Silico Evaluation

**DOI:** 10.3390/ijms241310959

**Published:** 2023-06-30

**Authors:** Mateusz Kciuk, Beata Marciniak, Ismail Celik, Enfale Zerroug, Amit Dubey, Rajamanikandan Sundaraj, Somdutt Mujwar, Karol Bukowski, Mariusz Mojzych, Renata Kontek

**Affiliations:** 1 Department of Molecular Biotechnology and Genetics, University of Lodz, Banacha 12/16, 90-237 Lodz, Poland; mateusz.kciuk@edu.uni.lodz.pl (M.K.);; 2 Doctoral School of Exact and Natural Sciences, University of Lodz, Banacha Street 12/16, 90-237 Lodz, Poland; 3 Department of Pharmaceutical Chemistry, Faculty of Pharmacy, Erciyes University, Kayseri 38280, Turkey; 4 Group of Computational and Pharmaceutical Chemistry, LMCE Laboratory, University of Biskra, BP 145, Biskra 07000, Algeria; 5 Computational Chemistry and Drug Discovery Division, Quanta Calculus, Greater Noida 274203, Uttar Prades, India; 6 Department of Pharmacology, Saveetha Dental College and Hospital, Saveetha Institute of Medical and Technical Sciences, Chennai 602105, Tamil Nadu, India; 7 Centre for Drug Discovery, Department of Biochemistry, Karpagam Academy of Higher Education, Coimbatore 641021, Tamil Nadu, India; 8 Chitkara College of Pharmacy, Chitkara University, Rajpura 140401, Punjab, India; 9 Department of Chemistry, Siedlce University of Natural Sciences and Humanities, 3 Maja 54, 08-110 Siedlce, Poland

**Keywords:** apoptosis, cytotoxicity, heterocycles, pyrazolo[4,3-*e*]tetrazolo[4,5-*b*][1,2,4]triazine, sulfonamides

## Abstract

Pyrazolo[4,3-*e*]tetrazolo[1,5-*b*][1,2,4]triazine sulfonamides (**MM**-compounds) are a relatively new class of heterocyclic compounds that exhibit a wide variety of biological actions, including anticancer properties. Here, we used caspase enzyme activity assays, flow cytometry analysis of propidium iodide (PI)-stained cells, and a DNA laddering assay to investigate the mechanisms of cell death triggered by the **MM**-compounds (**MM134**, **-6**, **-7**, and **-9**). Due to inconsistent results in caspase activity assays, we have performed a bromodeoxyuridine (BrdU) incorporation assay, colony formation assay, and gene expression profiling. The compounds’ cytotoxic and pro-oxidative properties were also assessed. Additionally, computational studies were performed to demonstrate the potential of the scaffold for future drug discovery endeavors. MM-compounds exhibited strong micromolar (0.06–0.35 µM) anti-proliferative and pro-oxidative activity in two cancer cell lines (BxPC-3 and PC-3). Activation of caspase 3/7 was observed following a 24-h treatment of BxPC-3 cells with IC_50_ concentrations of **MM134**, **-6,** and **-9** compounds. However, no DNA fragmentation characteristics for apoptosis were observed in the flow cytometry and DNA laddering analysis. Gene expression data indicated up-regulation of BCL10, GADD45A, RIPK2, TNF, TNFRSF10B, and TNFRSF1A (TNF-R1) following treatment of cells with the **MM134** compound. Moreover, in silico studies indicated AKT2 kinase as the primary target of compounds. **MM**-compounds exhibit strong cytotoxic activity with pro-oxidative, pro-apoptotic, and possibly pro-necroptotic properties that could be employed for further drug discovery approaches.

## 1. Introduction

More than seventy years ago, the cytostatic activity of N-mustard and its derivatives was discovered, marking the beginning of cancer chemotherapy. This finding paved the way for the synthesis of numerous antitumor molecules, including alkylating agents, antimetabolites, and antimitotics, which are effective against a wide range of human cancers [1]. The clinical efficacy of the many different anticancer treatments currently in use is diminished by drug resistance and the severe adverse effects of chemotherapeutic medications. As a result, there is perpetual demand for the discovery of novel or synergistic anticancer medications that produce fewer adverse effects [2]. Because of their prevalence in currently available pharmaceuticals, inherent diversity, and distinctive physicochemical features, heterocycle molecules have established themselves as true cornerstones of medicinal chemistry. Numerous agents are being studied that show promise against several cancers, in addition to those already on the market. Strategic inclusion of heterocyclic elements with certain physicochemical features is crucial for the design of compounds with high potency and selectivity [3,4].

There are many naturally occurring and synthetically produced physiologically active chemicals that contain the 1,2,4-triazine ring [5]. The pyrazolo[4,3-*e*][1,2,4]triazine ring system is a fascinating and understudied member of the group of 1,2,4-triazines, fused with a five-membered heterocycle that constitutes one of the most promising scaffolds for drug discovery. Many pyrazolo[4,3-*e*][1,2,4]triazines have exhibited anticancer activity in vitro. Specifically, tricyclic pyrazolo[4,3-*e*][1,2,4]triazines fused with a triazole or tetrazole ring have been found to possess cytotoxic and [6] and genotoxic activity [7,8] that result in the induction of apoptotic cell death [9,10,11].

Previous research has demonstrated that **MM129** (pyrazolo[4,3-*e*]tetrazolo1,5-*b*][1,2,4]triazine sulfonamide) efficiently limits cell viability by inhibiting Bruton’s tyrosine kinase (BTK) [10]. Furthermore, **MM129** exhibits antitumor activity in colon cancer xenograft mice. These results could be related to a decrease in serine/threonine-protein kinase AKT (AKT), cyclin-dependent kinase 2 (CDK2), mammalian target of rapamycin kinase (mTOR), and programmed death-ligand 1 (PD-L1) expression [12]. Here we have focused on the assessment of anticancer activity of four **MM129** analogues—**MM134**, **-6**, **-7,** and **-9** pyrazolo[4,3-*e*]tetrazolo[1,5-*b*][1,2,4]triazine sulfonamides. These **MM**-compounds exhibit cytotoxic and genotoxic activity in cancer cells in micromolar concentrations and show pro-apoptotic properties, as indicated by the externalization of phosphatidylserine (PS) on the outer plasma membrane of apoptotic cells, morphological changes explored with dual staining with acridine orange/ethidium bromide, and changes in the mitochondrial membrane potential (MMP; ΔΨm). These effects are selective for cancer cells compared with normal cells [8,11].

In the present study, we further explored the mechanisms of apoptotic cell death triggered in response to the incubation of the pancreas adenocarcinoma cell line (BxPC-3) and prostate adenocarcinoma cell line (PC-3) cells with the **MM**-compounds (**MM134**, **-6**, **-7** and **-9**) using caspase enzyme activity assays and flow cytometry analysis of cells stained with propidium iodide (PI). Due to the conflicting results obtained in the assays that estimate caspase activity, we performed a bromodeoxyuridine (BrdU) incorporation assay, colony formation assay, and gene expression profiling. The induction of reactive oxygen species (ROS) and their contribution to the cytotoxicity of the compounds were also evaluated. Additionally, computational studies were carried out to establish the primary target of the compounds based on the findings of **MM129** investigations, and to demonstrate the usefulness of this scaffold in further drug discovery approaches. The workflow of this study based on previous studies is presented in Figure S1 of the Supplementary Materials.

## 2. Results

### 2.1. Biological Studies

#### 2.1.1. Neutral Red Uptake Assay

A neutral red uptake assay was used to re-evaluate the cytotoxicity of **MM134**, **-6**, **-7**, and **-9** compounds following 24-h incubation with BxPC-3 and PC-3 cancer cells. The respective IC_50_ values obtained from two independent experiments with corresponding coefficients of determination (R^2^) are shown in Table S1 of the Supplementary Materials, together with the calculated mean IC_50_ values ± SD (standard deviation).

**MM**-compounds exhibited cytotoxic activity in BxPC-3 (IC_50_ range: 0.18–0.35 µM) and PC-3 (IC_50_ range: 0.06–0.17 µM) cancer cell lines following a 24-h incubation time. The PC-3 cell line was more prone to the cytotoxic activity of the compounds. **MM137** sulfonamide exhibited the highest cytotoxic potential in both cell lines, with IC_50_ values of 0.18 and 0.06 µM for the BxPC-3 and PC-3 cell lines, respectively.

#### 2.1.2. Reactive Oxygen Species (ROS) Formation

**Figure 1:**
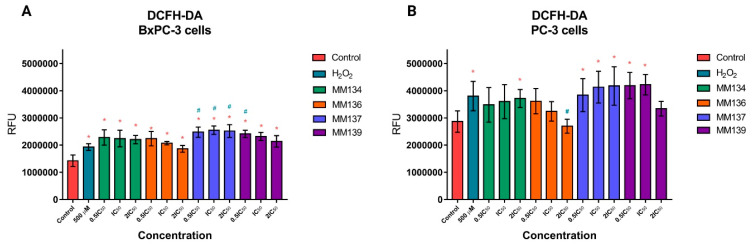
Induction of ROS in BxPC-3 (**A**) and PC-3 (**B**) cells following 1-h incubation with the **MM134**, **-6**, **-7**, and **-9** compounds in 0.5xIC_50_, IC_50_, and 2xIC_50_ concentrations, with hydrogen peroxide (H_2_O_2_) used as a positive control. The data are presented as relative fluorescence units (RFU) ± SD. The differences between the experimental samples and control samples were estimated using an ANOVA followed by Tukey’s post-hoc test (*p* < 0.05). * Significant difference compared with negative control; # Significant difference compared with positive control (500 µM H_2_O_2_).

Cellular ROS levels were estimated using a 2,7-dichlorodihydrofluorescein diacetate (DCFH-DA) fluorescent probe. After diffusion across the plasma membrane, 2′,7′-dichlorodihydrofluorescein (DCFH2) is generated by esterase cleavage of the acetate groups and is sequestered in the cytoplasm. Oxidation of DCFH2 leads to the generation of fluorescent 2′,7′-dichlorofluorescein (DCF, λ_ex_ = 503 nm, λ_em_ = 523 nm), which can be measured spectrofluorometrically. DCFH2 undergoes reactions with several different oxidants, although the reaction constants for each are different. Therefore, the probe is a great sensor for redox changes and general oxidative stress, as it reacts with multiple ROS including hydrogen peroxide, hydroxyl radicals, and peroxynitrite [13]. Induction of ROS in BxPC-3 and PC-3 cells following 1-h incubation with the **MM134**, **-6**, **-7**, and **-9** was estimated using DCFH-DA, as shown in Figure 1.

Following 1-h incubation of BxPC-3 cells (Figure 1A) with **MM134**, **-6**, **-7**, and **-9** compounds used in all tested concentrations (0.5xIC_50_, IC_50_, and 2xIC_50_), a statistically significant (*p* < 0.05) increase in ROS production compared with negative control was observed. A decrease in ROS production was observed with the increase in compound concentration for the **MM134**, **MM136**, and **MM139** compounds. **MM137** used in an IC_50_ concentration induced the highest increase in ROS production compared with other compounds, while the **MM136** compound used in a 2xIC_50_ concentration had the lowest pro-oxidative activity. A statistically significant increase in ROS production was shown for cells incubated with 500 µM H_2_0_2_ compared with the negative control. The increase in ROS production exceeded the pro-oxidative activity of 500 µM H_2_O_2_ used as a positive control for **MM137** in 0.5xIC_50_, IC_50_, and 2xIC_50_ concentrations, and **MM139** used in a 0.5xIC_50_ concentration (*p* < 0.05).

In the PC-3 cell line (Figure 1B), a statistically significant (*p* < 0.05) increase in ROS production (indicated as an increase in mean RFU) was observed following 1-h incubation of cells with **MM134** used in 2xIC_50_ concentration, **MM137** used in all tested concentrations (0.5xIC_50_, IC_50_ and 2xIC_50_), and **MM139** used in 0.5xIC_50_ and IC_50_ concentrations compared with the negative control. The **MM139** used in the above-mentioned concentrations induced the highest levels of ROS compared with the negative control. ROS production increased with the increase in compound concentration for the **MM134** and **MM137** compounds. No statistical significance was detected in comparisons to ROS levels of **MM**-treated cells with cells treated with 500 µM H_2_O_2_. Only the **MM136** derivative used in the 2xIC_50_ concentration induced statistically significant (*p* < 0.05) decreased levels of ROS compared with the positive control.

#### 2.1.3. Caspase 3/7/8/9 Detection

**Figure 2:**
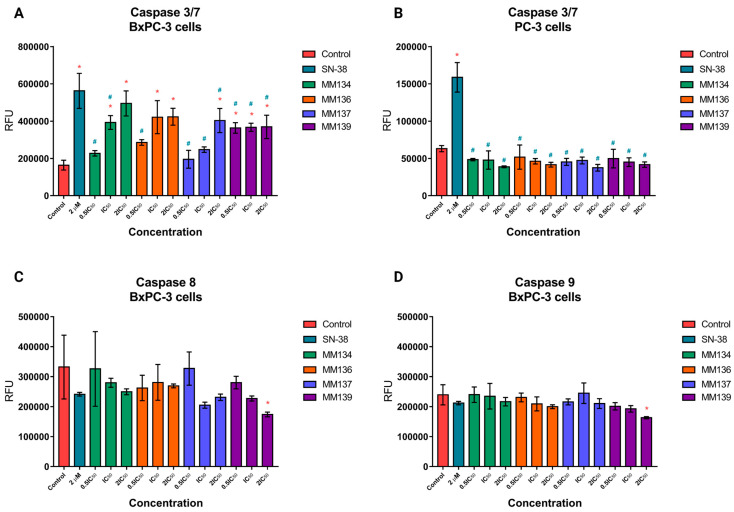
Caspase 3/7 activity in BxPC-3 (**A**) and PC-3 (**B**) cells following 24-h incubation with **MM**-compounds used at 0.5xIC_50_, IC_50_, and 2xIC_50_ concentrations and 2 µM SN-38 (used as positive control). Caspase 8 (**C**) and caspase 9 (**D**) activity in BxPC-3 cells treated with the same concentrations of the compounds and for the same incubation time. The data are presented as relative fluorescence units (RFU) ± SD. The differences between the experimental samples and control samples were estimated using an ANOVA followed by Tukey’s post-hoc test (*p* < 0.05). * Significant difference compared with negative control; # Significant difference compared with positive control (2 µM SN-38).

We have also previously shown that **MM134**, **-6**, **-7**, and **-9** compounds exhibit moderate DNA damage [8] and strong pro-apoptotic activity in both BxPC-3 and PC-3 cells, as indicated by mitochondrial membrane permeabilization (MMP), PS exposure on the cell surface, and differential uptake of fluorescent dyes—acridine orange/ethidium bromide (AO/EB) [11]. Here, we explored the influence of compounds on caspases enzymatic activity. The effect of 24-h incubation of BxPC-3 (Figure 2A) and PC-3 (Figure 2B) cells with **MM**-compounds (used in 0.5xIC_50_, IC_50,_ and 2xIC_50_ concentration) and 2 µM SN-38 (the active metabolite of camptothecin) on caspase 3/7 activity and caspase 8/9 activity for BxPC-3 cells (Figure 2C,D), respectively.

In BxPC-3 cells (Figure 2A), the **MM134** and **MM136** compounds used with the IC_50_ and 2xIC_50_ concentration induced statistically significant (*p* < 0.05) increases in caspase 3/7 activity (indicated as the increase in mean RFU) compared with the negative control. The **MM139** compound induced a statistically significant increase in caspase 3/7 activity in all tested concentrations (0.5xIC_50_, IC_50_, and 2xIC_50_) compared to the negative control. In contrast, the **MM137** compound induced a statistically significant increase in caspase 3/7 activity only in the 2xIC_50_ concentration compared to the negative control. The caspase 3/7 activation did not exceed the activity of 2 µM SN-38 in any of the experiments. To our surprise, **MM**-compounds in PC-3 cells (Figure 2B) did not induce a statistically significant (*p* < 0.05) increase in caspase 3/7 activity compared with the negative control. Only 2 µM of SN-38 induced a statistically significant increase in caspase 3/7 activity compared with the negative control.

Therefore, we decided to focus on the estimation of caspase 8/9 activity in BxPC-3 cells. In BxPC-3 cells (Figure 2C,D), only **MM139** used in the 2xIC_50_ concentration induced a statistically significant (*p* < 0.05) decrease in caspase 8/9 activity. In other experimental series and following incubation of cells with 2 µM SN-38, no statistically significant changes in caspase 8/9 activity were detected. The observed decrease in caspase activity in PC-3 cells, and in some cases in BxPC-3 cells (note that no statistically significant changes in caspase 8/9 activity were detected in BxPC-3 cells except at the 2xIC_50_ concentration of **MM139**), could be attributed to the decrease in cell proliferation. Therefore, we explored the effect of **MM**-compounds on PC-3 cell proliferation using a BrdU incorporation assay and a clonogenic assay.

#### 2.1.4. Bromodeoxyuridine (BrdU) Incorporation Assay

**Figure 3:**
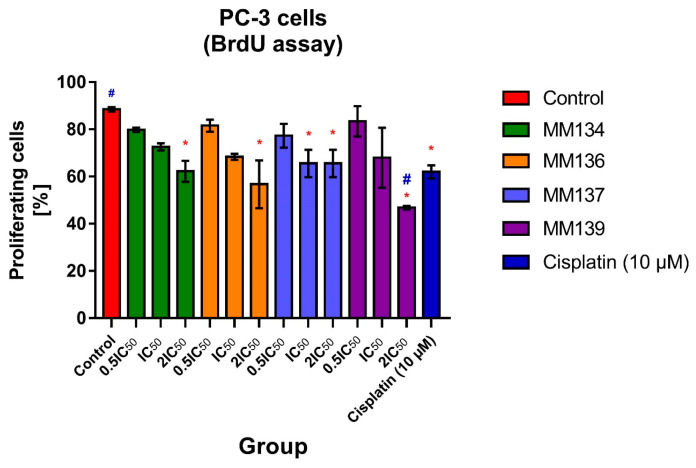
Effect of **MM**-compounds used in (0.5xIC_50_, IC_50_, and 2xIC_50_) concentrations and 10 µM cisplatin (used as positive control) on PC-3 cell proliferation following 24-h incubation. Data are presented as a mean percentage [%] of proliferating cells ± SD. An ANOVA followed by Tukey’s test was used to show statistically significant changes (*p* < 0.05) in the mean % of proliferating cells in samples compared with a negative control (indicated with *) and positive control (indicated with #).

The effects of **MM**-compounds used in 0.5xIC_50_, IC_50_, and 2xIC_50_ concentrations on PC-3 cell proliferation following 24-h incubation using a BrdU incorporation assay are shown in Figure 3. Additionally, 10 µM cisplatin was used in the experimental design as a positive control. The experiment was performed as a duplicate, and a total of at least 500 cells per experiment were counted using a fluorescence microscope. The data are presented as % of proliferating cells. The differences between the experimental samples and untreated control were evaluated using an ANOVA followed by Tukey’s test. A *p*-value less than 0.05 was considered statistically significant (*p* < 0.05).

**MM**-compounds used in the 2xIC_50_ concentration induced a statistically significant (*p* < 0.05) decrease in the mean % of proliferating cells in the tested samples compared with the negative control (% of proliferating cells = 88.5 ± 0.98). Incubation of PC3 cells with **MM137** compound used in the IC_50_ concentration also induced a statistically significant decrease in the mean % of proliferating cells (% of proliferating cells = 65.6 ± 5.79; *p* = 0.04). The **MM139** compound used in the highest (2xIC_50_) concentration induced the most profound reduction in cell proliferation (% of proliferating cells = 46.8 ± 0.77; *p* = 0.0002), and showed a superior effect compared to the 10 µM of cisplatin used in the experiment as a positive control (% of proliferating cells = 62 ± 2.8). The % of proliferating cells observed following the use of **MM**-compounds in the IC_50_ and 2xIC_50_ concentrations are shown in Table S2 of Supplementary Materials.

#### 2.1.5. Clonogenic Assay

The clonogenic assay, also known as the colony formation assay, is a type of in vitro cell survival assay that evaluates a single cell’s potential to form a colony. Cells are seeded out in appropriate dilutions either before or after treatment to establish colonies within one to three weeks. Colonies are counted after being fixed with glutaraldehyde (6.0% *v*/*v*), then stained with crystal violet (0.5% *w*/*v*) [14].

In the first step, the plating efficiencies were established by seeding different dilutions of cells. The examples of images obtained after seeding 100, 200, 500, 1000, and 2000 BxPC-3 or PC-3 cells per well of six-well plates are shown in Figure S2 of Supplementary Materials.

**Figure 4:**
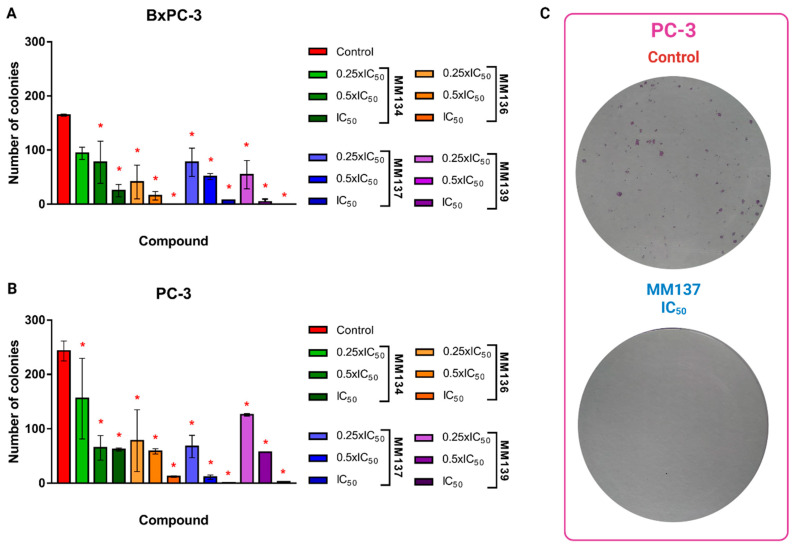
Effect of **MM**-compounds used in 0.25xIC_50_, 0.5xIC_50_, and IC_50_ concentrations on the clonogenic potential of BxPC-3 (**A**) and PC-3 (**B**) cells. (**C**) shows the effect of the IC_50_ concentration of the **MM137** compound on the clonogenicity of PC-3 cells compared with the control sample. Data are presented as the mean number of colonies ± SD. An ANOVA followed by Tukey’s test was used to show statistically significant changes (*p* < 0.05) between mean number of colonies in the samples (indicated with *). Created using BioRender.com, accessed on 24 April 2023.

To establish the optimal seeding numbers for BxPC-3 and PC-3 cells for the colony assay, we plated 100–2000 cells in six-well plates. The plating efficiencies were 25% per 100 cells, 26.25% per 200 cells, 27.4% per 500 cells, 25.85% per 1000 cells, and 17.8% per 2000 for BxPC-3 cells, and 37% per 100 cells, 25.3% per 200 cells, 37.3% per 500 cells, 31.6% per 1000 cells, and 24.45% per 2000. Therefore, we seeded 500 cells in six-well plates to test the effects of different concentrations of **MM** compounds on cell clonogenic ability. Figure 4 presents the effects of **MM**-compounds on the clonogenic potential of BxPC-3 (Figure 4A) and PC-3 (Figure 4B) cells, while Figure 4C shows the effect of the IC_50_ concentration of the **MM137** compound on clonogenicity of PC-3 cells compared with the control sample.

A statistically significant (*p* < 0.05) decrease in colony formation was observed following the incubation of cells with **MM**-compounds, both in BxPC-3 (Figure 4A) and PC-3 (Figure 4) cells.

In BxPC-3 cells, the **MM136** and **MM139** compounds exhibited the highest inhibitory effects on colony formation (mean numbers of colonies = 15.5 and 0 for cells incubated with thr 0.5xIC_50_ and IC_50_ concentrations of **MM136**, and 4 and 0 for cells incubated with **MM139** in the same concentrations, respectively).

In PC-3 cells, **MM137** and **MM139** exhibited the highest inhibitory potential, with mean numbers of colonies of 11 and 0.5 for **MM137** used in the 0.5xIC_50_ and IC_50_ concentrations, and 5.7 and 2.5 for **MM139** used in the same concentrations, respectively.

#### 2.1.6. Cell Cycle Analysis with Propidium Iodide (PI) Staining

During the cell cycle, the amount of DNA found in the parent cell rises because new DNA is synthesized in the S phase of the cell cycle. During the G2/M phase of the cell cycle, when there are two complete copies of the DNA, the cell divides into two new cells. Consequently, cells that are at various phases of the cell cycle contain varying amounts of DNA. A cell that is damaged or unable to complete the cell cycle is intended to die through apoptosis, protecting the integrity of the cell genome. As a direct consequence of this, apoptotic cells contain a lower proportion of DNA compared to live cells. Staining cells with propidium iodide (PI), a fluorescent reagent that intercalates with DNA, enables the measurement of the pro-apoptotic activity of compounds. The amount of DNA found in a cell has a direct and proportional relationship to the amount of PI fluorescence found in that cell [15,16].

**Figure 5:**
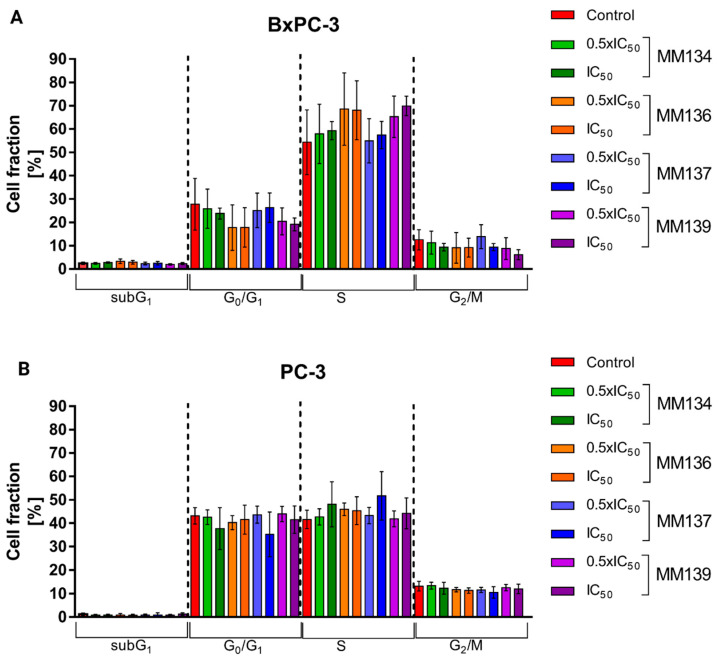
Effect of **MM**-compounds (**MM134**, **-6**, **-7** and -**9**) on the cell cycle of BxPC-3 (**A**) and PC-3 cells (**B**) following 24-h incubation of test with tested pyrazolo[4,3-*e*]tetrazolo[1,5,-*b*][1,2,4]triazine sulfonamides.

The effect of **MM**-compounds on the cell cycle of BxPC-3 and PC-3 cells is shown in Figure 5A,B, respectively.

A slight increase in the subG1 cell fraction (reflecting apoptosis induction) was observed following 24-h incubation of BxPC-3 cells with the IC_50_ concentration of **MM134** (2.8%), 0.5xIC_50_ and IC_50_ of **MM136** (3.3% and 2.9%), and IC_50_ of **MM137** (2.49%) compared with control cells (2.43%). However, no statistical significance (*p* < 0.05) was detected.

In contrast, the number of cells in the G0/G1 phase of the cell cycle seemed to decrease with the increase in **MM**-compounds concentration relative to the control, with an increase in cells in the S-phase of the cell cycle and a decrease in cells in the G2/M fraction, but no statistical significance was detected in these comparisons.

In the PC-3 cell line, an increase in the subG1 fraction was observed only for the IC_50_ concentration of **MM139** (1.31%) compared with the negative control (1.2%). However, this was not statistically significant (*p* < 0.05). Similar to BxPC-3 cells, a decrease in the G0/G1 and G2/M fractions and an increase in the S fraction was observed; however, no statistical significance (*p* < 0.05) was observed.

#### 2.1.7. DNA Laddering

**Figure 6:**
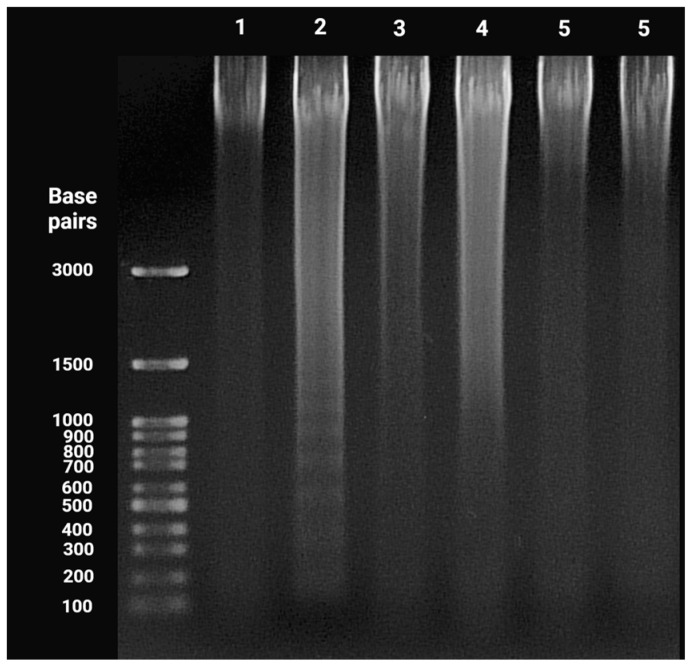
DNA fragmentation in BxPC-3 cells treated with the **MM134** (3), **MM136** (4), **MM137** (5), and **MM139** (6) compounds in their respective IC_50_ concentrations obtained using the neutral red uptake test. (1) Negative control: DMSO treated cells; (2) positive control: cells treated with 7-ethyl-10-hydroxycamptothecin (SN-38) in a 2 µM concentration.

Classical apoptotic cell death can be differentiated from other types of cell death by distinct morphological and biochemical characteristics. DNA fragmentation is a defining component of apoptosis. Endonucleases cleave DNA during apoptosis, culminating in the fragmentation of chromatin into nucleosomal components, which are multiples of approximately 180-bp oligomers. When the fragmented DNA is separated on an agarose gel, this distinctive DNA fragmentation may be seen as a ladder-like pattern [17,18]. BxPC-3 cells were used in this assay, as they are more responsive to the pro-apoptotic properties of the compounds [11]. However, similar to the cell cycle analysis (where no subG1 cell fraction was observed), no DNA fragmentation was detected. In contrast, DNA fragmentation was seen after the treatment of cells with an apoptosis-inducing agent: 7-ethyl-10-hydroxycamptothecin (SN-38) (Figure 6).

#### 2.1.8. Gene Expression Analysis

**Figure 7:**
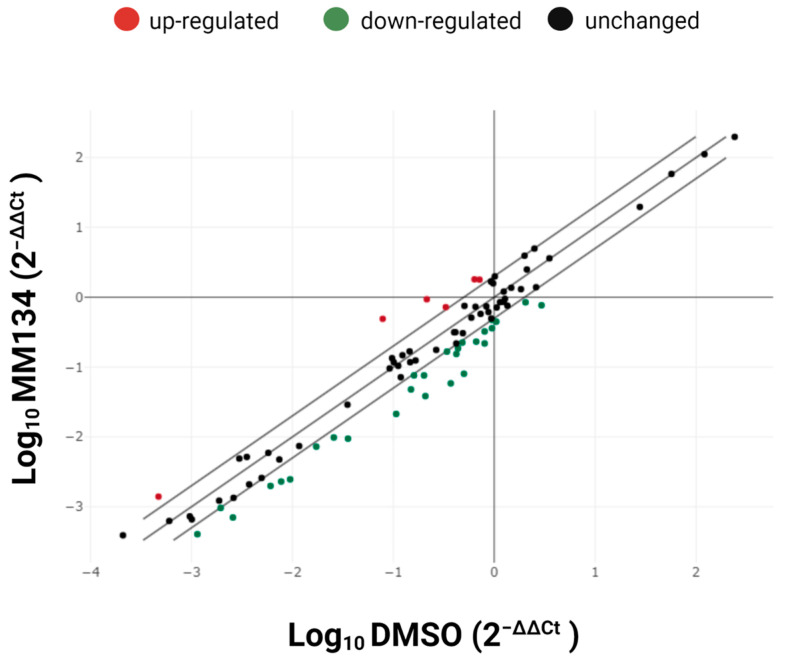
Differential gene expression in BxPC-3 cells following treatment with the **MM134** compound, expressed as a scatterplot of the 96 genes included in the current study.

To further explore the cell death pathway activated in response to **MM**-compounds, gene expression profiling using RT^2^ Profiler™ PCR array human apoptosis was performed. **MM134** compound was selected as the most pro-apoptotic of investigated compounds, as evidenced by PS exposure on the cell surface in our earlier investigations and the observation of morphological changes [11]. The impact of the IC_50_ concentration of **MM134** on BxPC-3 gene expression is shown in Figure 7. Gene expression differences between the control and treatment groups were considered significant with *p* < 0.05 and an absolute fold regulation > 2 as a cut-off. Upregulated genes are marked in red, and the downregulated genes are indicated in green. Fold-regulation is a biologically applicable representation of fold-change results. Fold-change values above one indicate upregulation of a gene, and the fold-regulation equals the fold-change. Fold-change values below one indicate downregulation of gene expression, and the fold-regulation represents the negative inverse of the fold-change.

A total set of 33 target genes were found to be differentially expressed (*p* < 0.05) (Table 1).

### 2.2. Computational Analysis

#### 2.2.1. Density Functional Theory (DFT) Calculations

Frontier orbital energies, such as the highest occupied molecular orbital (HOMO) and lowest occupied molecular orbital (LUMO), were estimated to obtain the electronic properties of the chemical molecules. LUMO directly corresponds to electron affinity and has the tendency to accept electrons, while HOMO depicts the ionization energy of a molecule [19]. The energy difference between HOMO and LUMO determines the chemical stability. A molecule with a low HOMO–LUMO energy gap (HLG) implies high polarizability, high reactivity, and low stability [20]. Based on the results, it was observed that the molecules (**MM134**, **MM136**, **MM137**, and **MM139**) displayed low HLG values. The HOMO and LUMO energy values ranged between −0.25 eV to −0.23 eV and −0.22 eV to −0.20 eV, indicating the fragile nature of the bound electrons. The energy gap values of the compounds varied between 0.04 eV and 0.01 eV. The calculated frontier orbital energy values for the compounds were tabulated (Table 2).

The electron distribution profile of the compounds is shown in Figure S3 of Supplementary Materials.

**Figure 8:**
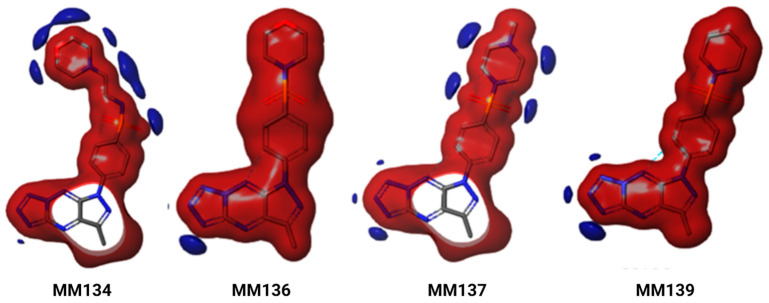
Molecular electrostatic potential (MESP) analysis. The electrophilic and nucleophilic reactive sites are represented as negative (red) and positive (blue) regions.

Figure S3 shows that both HOMO and LUMO orbitals are localized in two distinct parts of the molecules. HOMO map analysis of **MM134** showed that the electrons are located on the phenylsulfonyl group and the LUMO maps are localized on the pyrazole and triazine groups. In the case of **MM136**, the HOMO orbitals are widely dispersed around the morpholine region, whereas the LUMO is localized on the pyrazole group. The HOMO distributions for **MM137** and **MM139** were observed on the phenylsulfonyl and piperazine groups, while the LUMO was observed on the pyrazole, triazine (**MM137**), and phenylsulfonyl and piperazine groups (**MM139**). However, compared to other compounds, **MM139** showed increased HOMO and decreased LUMO energy values. In addition, **MM139** showed higher energy gap values (0.049 eV) compared to the other compounds. The energy distribution profile of HOMO and LUMO on the surface of the compounds provides information on the possible reactive sites. The low energy gap values obtained imply the high reactive nature of the compounds. In addition, molecular electrostatic potential (MESP) analysis was carried out to explore the reactivity and molecular bonding patterns in the compounds. The electrophilic and nucleophilic reactive sites are represented as negative (red) and positive (blue) regions, respectively (Figure 8).

In the case of **MM134** and **MM137**, the negative electrostatic potential (ESP) is localized on the entire molecule, except pyrazole and triazine regions, whereas the positive ESP is localized on the sulfur, nitrogen, and oxygen atoms of phenylsulfonyl, aminoethylmorpholine, and methylpiperazine groups. The MESP analysis of **MM136** and **MM139** indicates that both the molecules showed the deepest negative ESP for the entire molecules, with the positive ESP localized in the tetrazole region. The obtained results indicate the crucial sites that are responsible for the intermolecular and intramolecular interactions.

#### 2.2.2. Molecular Docking Studies of MM-Compounds

In the previous investigations, Hermanowicz et al. [12,21] explored the molecular basis of **MM129** compound anticancer activity. The authors declared that pyrazolo[4,3-*e*]tetrazolo[1,5-*b*][1,2,4]triazine sulfonamides can work as AKT, BTK, CDK2, mTOR, and PD-L1 inhibitors. However, this was based on the gene expression changes in colorectal cancer cells and did not evaluate the exact inhibitory effects of the compound on the proteins. In the initial in silico research, we performed molecular docking and molecular dynamics studies of the previously studied **MM129** compound and the compounds investigated in the present work (**MM134**, **-6**, **-7**, and **-9**). We found that compounds may inhibit the molecular targets to a similar or greater extent than previously investigated **MM**-compounds (including **MM129**) [8,11]. However, these results were established with the use of single molecular docking and dynamics software. Nevertheless, they allowed us to prioritize molecular targets for further in silico investigations. Therefore, in the present work, we included docking (with various scoring functions and conditions) and dynamics with previously unutilized dynamics software (Gromacs 2020.4). Performing in silico docking and dynamics studies using different software can be valuable for validation of the results, consensus prediction, and overcoming the limitations of a single software.

In the first step, molecular docking simulations were carried out to predict the molecular anticancer targets of the triazine sulfonamide analogs, and to evaluate the binding affinities and binding modes of these compounds with the predicted binding pockets of the target proteins. The Protein Data Bank (PDB) was mined for the 3D coordinates of AKT1 (PDBid = 3MVH) [22], AKT2 (PDB id = 3D0E) [23], BTK (PDBid = 3GEN) [24], serine/threonine-protein kinase CHK1 (CHK1) (PDBid = 2YM8) [25], mTOR1 (PDBid = 6BCX) [26], and PD-L1 (PDBid = 7BEA) [27] crystal structures, which originate from *Homo sapiens* organisms and exhibit high resolution. Hydrogen atoms were introduced to the protein using Discovery Studio 4.5 templates for protein residues. Hydration water molecules were kept or removed as required [28,29,30], and the protein structure was used in docking experiments without energy minimization. We performed the docking study using the Libdock docking engine [31], which treats the receptor as rigid and ligand molecules as flexible structures. Subsequently, high-ranking docking solutions were scored by seven different scoring functions (Jain [32], LigScore1, LigScore2 [33], PLP1, PLP2 [34], PMF, and PMF04 [35]). To determine the binding affinity between the ligand and receptor, we specifically considered the binding energy. The molecular docking simulation process for each anticancer target was validated by considering both chemical similarity and the superposition of the docked ligand conformation with its co-crystallized conformation [36,37]. Following this, we utilized similar parameters to perform simulation studies on the triazine sulfonamide analogs.

Table 3 displays the four anticancer targets (AKT2, BTK, CHK1, and PD-L1) for which the docked pose of triazine sulfonamide derivatives (**MM134**, **MM136**, **MM137**, and **MM139**) exhibited a high binding affinity. These four targets were selected from the previously identified six targets. The results presented in Table 3 were obtained using precise docking conditions. The optimal outcomes were obtained using the Ligscore 2 scoring function for BTK, CHK1, and PD-L1, while the Ligscore 1 scoring function was utilized for the AKT2 target. Moreover, the **MM134**, **-6**, **-7**, and **-9** compounds were docked into the hydrous binding site of the CHK1 and BTK targets and the anhydrous binding pocket of the AKT2 and PD-L1 targets.

**Figure 9:**
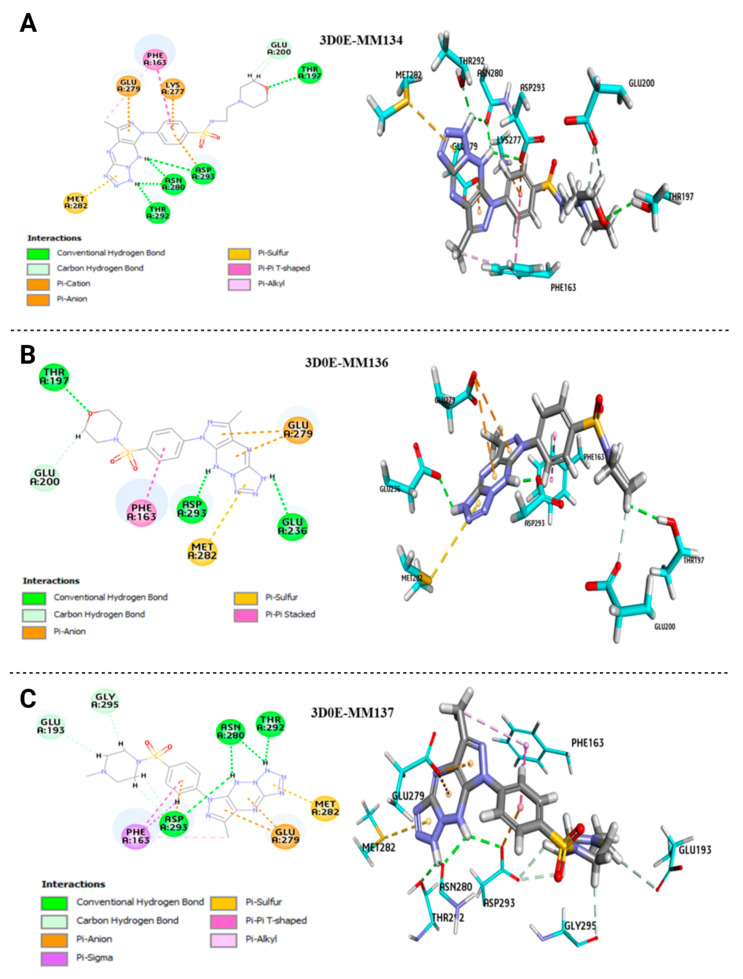
Binding interactions of **MM134** (**A**), **MM136** (**B**), and **MM137** (**C**) compounds with the crystal structure of AKT2 (3D0E). The 2D/3D interaction plots are represented on the left and right, respectively. The legends for the type of interactions are provided in respective 2D plots.

The **MM134**, **MM136**, **MM137**, and **MM139** compounds exhibited better binding energy (BE) scores with AKT2 and BTK targets. The sulfonamide derivatives **MM134**, **MM136**, and **MM137** exhibited higher binding energy scores when docked into the anhydrous binding pocket of AKT2, with binding energy scores of −70.158, −96.359, and −52.722, respectively. In contrast, **MM139** showed high-affinity to the hydrous binding site of BTK, with a BE score of −48.185. These results are consistent with our previous studies on these triazine sulfonamide analogs [8,11], which suggested that the compounds’ apoptotic potential may arise from their ability to inhibit the activity of AKT2, BTK [11], CHK1 [8], and PD-L1 [11] (Table 4). Figure 9 displays the 2D/3D interaction plots of AKT2’s active binding pockets (PDB:3D0E) with the **MM134**, **-6**, and **-7** compounds.

These plots revealed a shared agreement among the **MM134**, **MM136**, and **MM137** compounds, which were docked accurately, in placing the NH atoms of triazine and tetrazole groups close to the carbonyl groups of Asn280, Asp293, and Thr292 amino acids of AKT2 through the formation of hydrogen bond interactions in these positions. The **MM136** compound exhibited a similar interaction pattern, but only with the carbonyl groups of the Asp293 and Glu236 amino acids.

The oxygen atom of the morphine fragment of the **MM134** and **MM136** ligands forms conventional hydrogen bond interactions with the carbonyl groups of the Thr197 amino acid in the protein structure. **MM134** and **MM136** showed a similar binding pattern, where H atoms of the morpholine formed hydrogen bond interaction with Glu200:O amino acids of the protein. Similarly, the **MM137** compound exhibited the same interaction, but with the Gly295:O and Glu193:O amino acids.

When docked, the **MM134**, **MM136**, and **MM137** compounds shared the same binding feature, where tetrazole rings interacted with the Met282:S amino acid. This dictated a π–sulfur interaction at this position. The benzene of the phenylsulfonyl group in the **MM134**, **MM136**, and **MM137** compounds established a π–π interaction with the hydrophobic side chain of Phe163. Furthermore, the latter formed a π–alkyl interaction with the alkyl functional group of pyrazole in the **MM134** and **MM137** compounds. The residue Glu279:O formed a π–anion interaction with the tetrazole and triazine groups of the **MM136** and **MM137** compounds, as well as with the pyrazole fragment of the **MM134** compound.

The binding mode of triazine sulfonamide derivatives (**MM134**, **MM136**, and **MM137** compounds) involved the same residues in AKT2, namely Asn280, Asp293, Thr292, Thr197, Met282, Phe163, Glu279, Glu36, Glu200, Gly295:O, and Glu193. There is evidence in the literature to suggest that the activity of AKT2 may indeed depend on the presence of these residues [38,39]. The binding mode of the **MM134** and **MM137** compounds in AKT2 offers valuable insights into the binding and inhibition of AKT2 by these compounds. Nevertheless, the **MM139** compound exhibited a different binding mode with AKT2 compared with the other **MM**-compounds, with different amino acids interacting than those for the **MM134**, **MM136**, and **MM137** compounds. This disparity in binding mode could potentially explain why the **MM139** ligand has a lower binding energy with the AKT2 but has a higher binding affinity with the BTK.

**Figure 10:**
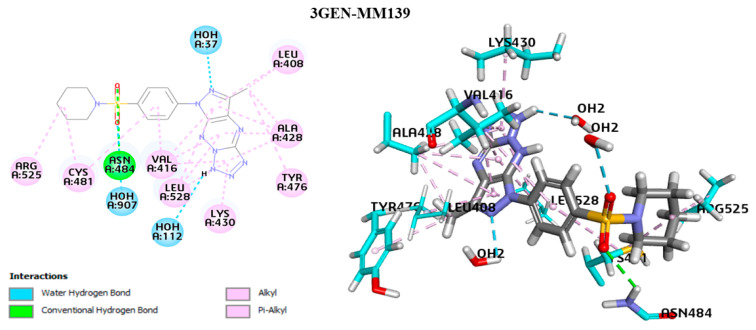
Binding interactions of the **MM139** compound with a hydrous binding pocket of BTK (3GEN). The 2D/3D interaction plots are represented on the left and right, respectively. The legends for the type of interactions are provided in respective 2D plots.

The 2D/3D interaction plots of the **MM139** compounds with a hydrous binding pocket of BTK (3GEN) are displayed in Figure 10.

It was found that the entire structure of **MM139** was favorably located in the BTK pocket. The carbonyl of the **MM139** compound’s sulfone fragment formed a conventional hydrogen bond with the Asn484:HD22 amino acid. Furthermore, the π–alkyl interaction is established with Leu408, Tyr476, Ala428, Leu528, Cys481, Lys430, and Val416 of BTK and triazine, tetrazole, and pyrazole fragments. These amino acids constitute a well-established active binding pocket for BTK kinase [40]. SKS151, a known BTK inhibitor, was previously found to engage in hydrophobic interactions with Leu408, Val416, and Leu528 [23]. Furthermore, the alkyl’s pyrazole and pyridine fragments generated alkyl interactions with Ala428, Cys481, Arg525, and Leu408. The **MM139** compound formed hydrogen bonds with water molecules, HOH37:H1, HOH907:H2, and HOH112:OH2. The presence of water molecules is crucial in accurately predicting ligand-protein docking. This is because water molecules can act as a bridge, linking the protein and the ligand to stabilize the complex. It is essential to explicitly include water molecules in the binding process, as demonstrated by the 3GEN–**MM139** complex, which required the presence of binding site hydration molecules to make reasonably accurate predictions. The higher BTK inhibition of compound 14G in the study of Zhao et al. resulted from the formed hydrogen bond interaction with the water molecule, which was similar to the co-crystallized molecule B43 of the BTK (PDB code 3GEN) target [41]. Thus, it is important to consider the role of water molecules in ensuring the accuracy of ligand–protein docking predictions [28,29]. We have focused on the AKT2 enzyme in further research, given its involvement in the pathophysiology of solid tumors, in contrast to the BTK enzyme, which is involved mainly in the development of B-cell malignancies [42].

#### 2.2.3. Prime MM-GBSA Calculations

The binding efficacies of ligands docked into the binding site of AKT2 protein were rescored using prime MM-GBSA. The calculated free energy of binding and free energy components for the protein–ligand complexes are shown in Table 4. The results show that the four complexes showed a binding energy value within the range of −46.50 kcal/mol to −25.15 kcal/mol. The non-polar solvation (ΔG_solv_), polar solvation (ΔG_solvlipo_), van der Waals, and coulomb energy were the driving forces for the ligand binding into the protein active site. The compound **MM139** disfavored the covalent binding, as evident from its low covalent binding value compared with the other complexes.

**Table 4:** Binding free energy calculation for the protein–ligand complexes.

Compound	ΔG_coulomb_ ^a^	ΔG_vdw_ ^b^	ΔG_covalent_ ^c^	ΔG_solv_ ^d^	ΔG_solvlipo_ ^e^	ΔG_bind_ ^f^
**MM134**	91.46	−46.61	4.15	−76.13	−19.14	−46.50
**MM136**	159.19	−40.97	6.37	−150.91	−16.10	−45.90
**MM137**	119.93	−44.90	7.51	−91.23	−16.14	−25.15
**MM139**	153.58	−42.90	1.23	−134.11	−18.26	−41.19

^a^ Contribution to the MM-GBSA free energy of binding from the coulomb energy. ^b^ Contribution to the MM-GBSA free energy of binding from the van der Waals energy. ^c^ Contribution to the MM-GBSA free energy of binding from the covalent binding. ^d^ Contribution to the MM-GBSA free energy of binding from the nonpolar contribution to the solvation energy due to the surface area. ^e^ Contribution to the MM-GBSA free energy of binding lipophilic binding. ^f^ Free energy of binding.

#### 2.2.4. Molecular Dynamics of MM-Compounds–AKT2 Complexes

**Figure 11:**
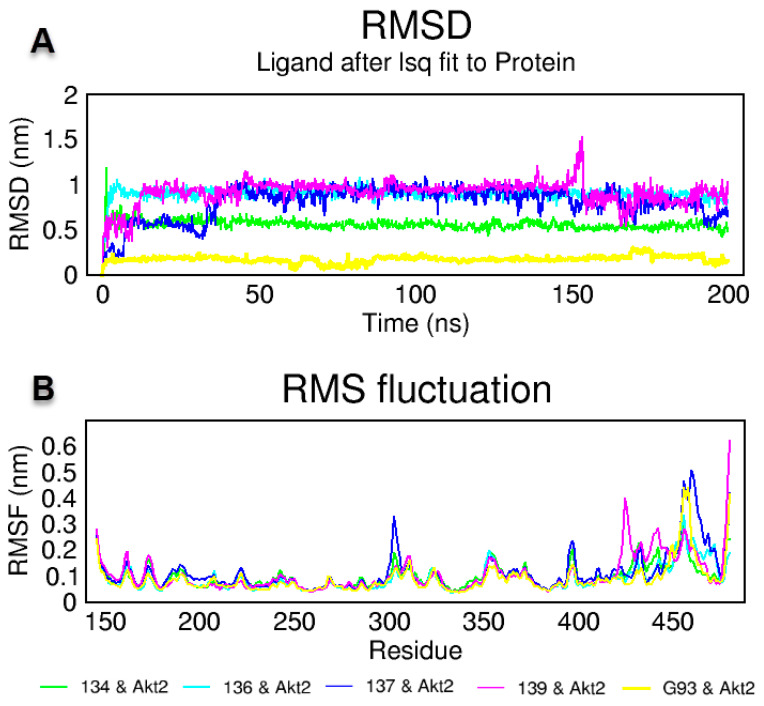
Trajectory analysis of complexes of the compounds **MM134**, **MM136**, **MM137**, **MM139**, and cocrystal ligand G39 with AKT2. Root mean square deviation (RMSD) (**A**) and root mean square fluctuation (RMSF) (**B**) of **MM134**–AKT2, **MM136**–AKT2, **MM137**–AKT2, **MM139**–AKT2, and G39–AKT2 protein–ligand complexes for 200 ns.

Molecular dynamics simulations are very useful in the drug research process to predict the stability of protein–ligand complexes in the in silico physiological environment [43]. In this study, the stability of **MM134**, **MM136**, **MM137**, and **MM139** with AKT2 (PDB ID: 3D0E) and its native co-crystalized ligand G39 were investigated using an MD simulation [44]. An MD simulation of 200 ns duration was performed. Trajectory root mean square deviation (RMSD) and root mean square fluctuation (RMSF) analyses were performed. RMSD is the basic parameter that numerically expresses the shifts of protein and ligand atoms [45]. As presented in Figure 11A, complexes of **MM134** and **MM136** with AKT2 remained stable after the first 5 ns at 0.6 nm and 1 nm, respectively. The complex of **MM137** with AKT2 started at 0.2 nm and increased to 0.5 nm in the first 10 ns, then it remained stable for a certain period. It increased to 1 nm after 40 ns and stabilized at 1 nm. The complex of **MM139** with AKT2 remained stable close to 0.5 nm for the first 15 ns, then increased to 1 nm and stabilized.

RMSF measurements were performed to show the fluctuation and mobility per residue in the protein structure [46]. As provided in Figure 11B, complexes of **MM139** and co-crystalized G39 with AKT2 produced high fluctuations. All AKT2 complexes showed a similar fluctuation trend around active site amino acids 150–300, starting at 0.3 nm and decreasing to 0.1 nm. Finally, animation videos from 285 frames recorded during 200 ns at the AKT2 active site of the compounds were created, as given in the Supplementary Materials for visual inspection. An MD simulation animation of 285 snapshots between 0 and 200 ns was generated.

In addition, AKT2–**MM134**, **-6**, **-7**, **-9**, and G39 protein–ligand interactions at 0 and 200 ns are provided in Figures S4–S6 of Supplementary Materials.

As shown in Figure S4A,B, the van der Waals interaction of the AKT2–**MM134** complex with Gly295, Leu183, Glu193, Gly159, Thr162, and Leu296 was preserved, but the interaction with Lys181 had transformed to a hydrophobic interaction from van der Waals interactions in the 0 and 200 ns MD simulation. Despite this, the complex remained stable. As shown in Figure 11A, in the AKT2–**MM134** complex, the RMSD value was measured below 1 nm and did not increase during the 200 ns MD simulation of this compound. Second, as given in Figure S4C,D, the π–sulfur interaction with Met282 transformed to a π–alkyl interaction at the end of 200 ns in the AKT2–**MM136** complex, while the ligand remained stable. As shown in Figure 11A, in contrast, in the AKT2–**MM136** complex, the RMSD value of **MM136** was calculated to be below 1 nm. As given in Figure S5A,B, interactions with Met282 continued in the AKT–**MM137** complex, but it had transformed to a π–alkyl interaction from the π–sulfur interactions. The AKT–**MM139** complex had preserved interaction between Lys160, Lys181, and Phe163, but all these interactions transformed at the end of the 200 ns simulation. Lys160 had shown hydrophobic interactions in 0 ns, and at the end of 200 ns, this interaction had transformed into van der Waals interactions. The interaction with Lys181 had transformed into a hydrophobic interaction from van der Waals interactions (Figure S5C,D). The RMSD value of AKT–**MM139** complexes produced high fluctuations between 0.5 nm to 1.5 nm (Figure 11A). Finally, protein–ligand interactions in the AKT–G39 complex were analyzed. As shown in Figure S6A,B, G39’s donor–donor interaction with Ala232 and hydrophobic interaction with Glu230 and Ala232 remained stable. For G39, hydrophobic interactions with Val166, Phe294, Phe439, Ala179, and Lue158 were preserved (Figure S6A,B). As shown in Figure 11A, the RMSD value of G39 in the AKT2–G39 complex was calculated to be below 0.5 nm.

Another way to measure protein–ligand stability and potency is to measure the number of H bonds formed between the protein and ligand [47]. In this context, the number of H bonds formed by compounds **MM134**, **MM136**, **MM137**, **MM139,** or a co-crystallized ligand with AKT2 for 200 ns was calculated, as provided in Figure S7A–E of Supplementary Materials. Compound **MM134** usually formed two, sometimes three H bonds with the AKT2 enzyme, while **MM136** established two H bonds. In contrast, **MM137** formed three to four H bonds up to 150 ns, and one H bond after 150 ns. The G93 native (co-crystalized) ligand formed five to seven H bonds with the protein.

One of the most important methods of measuring protein compactness is to measure the Rg value [48]. The Rg value of protein–ligand complexes was calculated, as shown in Figure S7F–J. AKT2–**MM134**, **MM136**, **MM137**, and **MM139** complexes established an Rg value of approximately 2.15 nm, while the AKT2–G93 complex produced an Rg value of approxiamtely 2.1 nm.

The determination of binding energies is crucial for understanding the strength of molecular interactions in protein–ligand complexes [49]. In this study, we examined the binding energies of AKT2 complexes with **MM134**, **MM136**, **MM137**, and **MM139**, and compared them to the AKT2–G39 complex. The binding free energies were calculated using molecular mechanics models, and van der Waals, electrostatic, polar solvation, and SASA contributions were considered, as given in Table 5. Our analysis aimed to assess the relative strengths of binding within the AKT2 complexes and discern their disparities concerning the AKT2–G39 interaction.

The comparison of binding energies among the AKT2 complexes revealed notable differences. Among the complexes studied, AKT2–**MM136** exhibited the most negative binding free energy, indicating a stronger binding affinity than the other complexes. Following this, AKT2–**MM137** and AKT2–**MM139** displayed the second and third-most negative binding energies, respectively. In contrast, AKT2–**MM134** had the least negative binding free energy, suggesting a weaker binding propensity compared to the other complexes.

Further comparison of the binding energies with the AKT2–G39 complex elucidated additional insights. AKT2–**MM136** exhibited a less negative binding free energy than the AKT2–G39 complex, signifying a relatively weaker binding interaction in comparison. Similarly, AKT2–**MM134,** AKT2–**MM137**, and AKT2–**MM139** also demonstrated less negative binding energies than the AKT2–G39 complex.

The investigation of binding energies in AKT2 complexes with **MM134**, **MM136**, **MM137**, **MM139**, and the G39 complex provided valuable insights into their relative binding strengths. The analysis indicated that AKT2–**MM136** exhibited the strongest binding affinity among the complexes studied, while AKT2–**MM134** displayed the weakest binding propensity. Furthermore, a comparative assessment with the AKT2–G39 complex demonstrated that all the other complexes (**MM136**, **MM137**, and **MM139**) exhibited weaker binding energies. These findings contributed to a better understanding of the differential binding characteristics within the AKT2 complexes and their deviations from the reference interaction.

## 3. Discussion

Purines are the most common and versatile N-heterocyclic chemicals that can be found in nature. Purine is the ideal scaffold for the discovery of innovative therapeutic medicines that target selectively purine-dependent enzymes and receptors. Many drug development efforts have focused on structural alterations of natural purines, especially those involving isosteric ring structures. Seven bicyclic heterocyclic systems isosteric to purine have been produced by fusing the 1,3,5-triazine ring with pyrrole, pyrazole, imidazole, 1,2,3-triazole, or 1,2,4-triazole [50]. The scaffold 1,2,4-triazine is less-known. The 1,2,4-triazine molecule is one of the three potential isomers of the six-membered ring that contains three nitrogen atoms. The 1,2,4-triazine ring is thought to be essential for a variety of different pharmacological actions, including anticancer activity. Therefore, numerous heterocyclic components were fused with this scaffold to enhance the antitumor activity. Pyrrolo[2,1-*c*][1,2,4]triazine and pyrrolo[2,1-*f*][1,2,4]triazine constitute the best examples of triazine compounds with antitumor activities [5,51]. In contrast to pyrrolotriazines, the pyrazolo[4,3-*e*][1,2,4]triazine ring system has been less studied. Because early studies on the group of simple substituted pyrazolotriazine derivatives did not show their considerable anticancer activities, it was decided to combine it with different pharmacophore groups, including sulfonamide moieties. Representatives of this class of compounds exhibited antitumor activity through inhibition of ABL kinase [52], carbonic anhydrases (CAs) [53,54], and CDKs, as previously reviewed [6]. Pyrazolo[4,3-*e*][1,2,4]triazines condensed with 1,2,4-triazole or a tetrazole ring constitute a particularly interesting group of compounds with anticancer activities in the nano and micromolar range [6].

More recently, a novel compound known as **MM129** (pyrazolo[4,3-*e*]tetrazolo[1,5-*b*][1,2,4]triazine sulfonamide) was shown to effectively limit cell viability by inhibiting the BTK protein [10]. BTK is a nonreceptor tyrosine kinase involved in B-lymphocyte development, differentiation, and signaling. The activation of B-cell antigen receptor signaling in secondary lymphatic organs triggers the excessive proliferation of malignant B cells. Over the past decade, BTK inhibitors have become an increasingly popular alternative to chemotherapy-based regimens, particularly in patients who suffer from chronic lymphocytic leukemia (CLL) and mantle cell lymphoma (MCL) [55,56]. **MM129** also exhibited anticancer potential in vivo in colon cancer xenograft mice. This effect might be attributed to the decrease in the expression of AKT, CDK2, mTOR, and PD-L1 [12]. The experimental results obtained for **MM129** are in line with the results of molecular docking and molecular dynamics simulation, where **MM129** and pyrazolo[4,3-*e*]tetrazolo[1,5-*b*][1,2,4]triazine sulfonamide derivatives (**MM131**, **-4**, **-6**, -**7** and **-9**) exhibited inhibitory effect on multiple CDKs, AKT, BTK, mTOR, and PD-L1 [11].

It was shown that **MM131** triggered apoptosis of DLD-1 and HT-29 cells with observed down-regulation of mTOR kinase, soluble intercellular adhesion molecule-1 (sICAM-1), or cathepsin [57]. **MM129** and **MM131**, together with the third sulfonamide derivative of pyrazolo[4,3-*e*]tetrazolo[1,5-*b*][1,2,4]triazine (**MM130**), exhibited cytotoxic and genotoxic activity, as established using an alkaline/neutral comet assay and γ-H2AX staining in four cancer cell lines: HeLa, HCT-116, PC-3, and BxPC-3. **MM129**, **MM130,** and **MM131** showed cytotoxic activity with IC_50_ concentrations of IC_50_ = 0.17–1.15 μM, as indicated by an MTT assay following 72-h incubation of the four above-mentioned cancer cell lines with the compounds [7].

The compounds investigated in this study (**MM134**, **-6**, **-7**, and **-9**) exhibited micromolar (IC_50_ range: 0.11–0.33 µM) cytotoxic activity in BxPC-3 and PC-3 cancer cells in the same assay, while showing minor cytotoxic activity in human normal lung fibroblasts (WI-38) (where IC_50_ values varied between 0.27–0.65 µM). The genotoxic activity of the compounds in the comet assay and γH2AX staining was also described. Furthermore, it was indicated that the DNA-damaging capability of the compounds may be attributed to the inhibition of CHK1 kinase, as indicated by in silico results [8,11]. Furthermore, these compounds exhibited profound pro-apoptotic activity in the BxPC-3 cell line following 24 and 48 h incubation of cells in vitro, where 2xIC_50_ concentrations of all **MM**-compounds induced apoptosis of 68.1 ± 7.33% to 95.1 ± 1.48% of the cells [11]. In contrast, Gornowicz et al. investigated the effect of 24-h incubation of **MM137** in DLD-1 and HT-29 cells. **MM137** exhibited cytotoxic activity, with IC_50_ values of 0.43 µM in DLD-1 cells and 0.16 µM in HT-29 cells [9]. Similar to the results obtained by our group [11], the authors indicated cancer-cell-specific cytotoxic activity of the compound. The cell viability of fibroblasts was reduced by 13.72% following 24-h incubation with **MM137** at a concentration of 0.5 µM [9]. **MM137** used in the same concentration induced apoptosis of colorectal cancer cells. The number of apoptotic cells was indicated as 68.6% for DLD-1 and 38.1% for HT-29 cells using the FITC-Annexin-V binding assay. The authors also detected a decrease in mitochondrial membrane potential (MMP) following treatment with the **MM137** compound, indicating apoptosis induction [57]. We also found changes in MMP following 24 and 48-h treatment of BxPC-3 and PC-3 cells with the **MM134**, **-6**, **-7**, and **-9** compounds [11]. The cytotoxicity of the pyrazolo[4,3-*e*]tetrazolo[1,5-*b*][1,2,4]triazine sulfonamide compounds (Table S3) is supported by literature data, and the possible molecular mechanism of **MM**-compounds activity, as indicated by the literature, is presented in Figure S8 of the Supplementary Materials.

In this study, we confirmed the cytotoxic activity of **MM**-compounds using a neutral red uptake assay. The investigated compounds exhibited cytotoxic activity in BxPC-3 (IC_50_ range: 0.18–0.35 µM) and PC-3 (IC_50_ range: 0.06–0.17 µM) cancer cell lines following a 24-h incubation time. The PC-3 cell line was more prone to the cytotoxic activity of the compounds. Consistent with previous results [11], **MM137** sulfonamide exhibited the highest cytotoxic potential in both cell lines, with IC_50_ values of 0.18 and 0.06 µM for the BxPC-3 and PC-3 cell lines, respectively. Furthermore, to confirm apoptosis induction following treatment with the **MM**-compounds, we evaluated the caspase activity in BxPC-3 and PC-3 cells. In the BxPC-3 cells (Figure 2), the **MM134** and **MM136** compounds in IC_50_ and 2xIC_50_ concentrations induced an increase in caspase 3/7 activity compared with the negative control. The **MM139** compound induced a statistically significant increase in caspase 3/7 activity across all tested concentrations (0.5xIC_50_, IC_50_, and 2xIC_50_) compared to the negative control. In contrast, the **MM137** compound induced a statistically significant increase in caspase 3/7 activity only in the 2xIC_50_ concentration compared to the negative control. Induction of caspases activity was also previously observed following 24-h treatment of DLD-1 and HT-29 cells to **MM131** [57] and **MM137 [9]**.

No increase in caspase 3/7 activity was observed for PC-3 cells (Figure 2B). This was consistent with the lower pro-apoptotic activity of the compounds in PC-3 cells following 24-h treatment with the tested compounds that were obtained in our previous studies [11]. Surprisingly, we did not observe caspase-8/9 induction in BxPC-3 cells treated with the **MM134**, **-6**, **-7**, and **-9** compounds (Figure 2C,D). This could be attributed to the decrease in the proliferation of cells in the caspase activity assay, which could affect the levels of caspases in each well. Based on the decrease in caspase 3/7 activity in PC-3 cells, we chose this cell line for further investigation. Using a BrdU incorporation assay, we evaluated the effect of **MM**-compounds on cell proliferation. We found cytostatic activity of the investigated compounds, which was especially profound for the **MM139** compound used at the 2xIC_50_ concentration (% of proliferating cells = 46.8 ± 0.78%). These results are consistent with the lowest activity of caspase enzymes observed following the treatment of BxPC-3 and PC-3 cells with this compound. **MM**-compounds also dramatically decreased the clonogenic potential of both BxPC-3 and PC-3 cells, further explaining the obtained results and suggesting that the compounds exhibited more cytostatic than cytotoxic activities.

Following 1 h incubation of BxPC-3 cells and PC-3 cells, an increase in the overall ROS production was observed (Figure 1). The dose-dependent decrease of the fluorescence found in cells incubated with tested compounds could be attributed to the same reason that was indicated for the caspase assays. However, an increase in ROS production in the DCFH-DA assay confirmed the oxidative stress generated by compounds in the studied cell lines. In contrast, no changes in the cell cycle distribution were found after the treatment of BxPC-3 and PC-3 cells with compounds used in 0.5xIC_50_ and IC_50_ concentrations (Figure 5).

Despite the negative results obtained during the 24-h cell cycle analysis, apoptosis induction in cells following treatment with **MM**-compounds was not excluded. The presence of a hypodiploid DNA peak is not proof of apoptotic cell death. The exposed cell can be negative for the sub-G1 peak, as DNA fragments are still retained in the nucleus [15]. Moreover, the presence of DNA strand breaks following incubation with **MM**-compounds was confirmed through the alkaline/neutral comet assay and γ-H2AX staining [7,8]. To further explore apoptosis induction, we performed a DNA fragmentation assay. No DNA laddering was observed following the treatment of cells with IC_50_ concentrations of the **MM134**, **-6**, **-7**, and **-9** compounds. These findings, together with the absence of observed caspase activation (besides caspase-3/7 induction) and the exposure of PS on the cell surface, imply the induction of necroptosis in cells, and not apoptosis. However, observation of apoptotic bodies, especially following prolonged (48-h) incubation of cells with compounds, could also indicate the activation of late stages of apoptosis [9,11,57]. The activation of caspases 3/7 following cancer cell treatment was observed not only in BxPC-3 cells treated with **MM134**, **-6**, **-7**, and **-9** compounds, but also other derivatives investigated by other authors, confirming the pro-apoptotic potential of the pyrazolo[4,3-*e*]tetrazolo[1,5-*b*][1,2,4]triazine sulfonamides. A summary of cellular response to MM-compounds is presented in Figure S9 of Supplementary Materials.

The upregulation of five genes was shown in the gene expression analysis of BxPC-3 cells treated with the **MM134** compound. These included B-cell CLL/lymphoma 10 (BCL10), growth arrest and DNA-damage-inducible, alpha (GADD45A), receptor-interacting serine-threonine kinase 2 (RIPK2), tumor necrosis factor (TNF), tumor necrosis factor receptor superfamily, member 10b (TNFRSF10B) and tumor necrosis factor receptor superfamily, and member 1A (TNFRSF1A; TNF-R1) (Table 1).

BCL10 is a protein from the caspase recruitment domain (CARD) family that regulates apoptosis and the nuclear factor NF-kappa-B (NF-κB) signaling pathway [58]. Overexpression of BCL10 in cell cultures has been shown to promote apoptosis [59]. The CARD domain present at BCL10 amino-terminal domain is thought to mediate the binding of adapter molecules and caspases. These CARD-containing proteins interact with one another in response to a range of stressors, including DNA-damaging agents and cell-death ligands like FAS and TNF. The resulting binding activates downstream signaling, triggering apoptosis [59]. However, BCL10 also participates in the formation of complexes that inhibit apoptosis and is important for cell survival following DNA damage. Cytoplasmic BCL10 translocates to the nucleus to facilitate DNA damage repair, including histone ubiquitination and the accumulation of homologous recombination (HR) repair components [60].

TNFRSF10B (also known as death receptor 5 (DR5)) belongs to the TNF-receptor superfamily and has an intracellular death domain. When stimulated by tumor necrosis factor-related apoptosis-inducing ligand (TNFSF10/TRAIL/APO-2L), this receptor transmits an apoptotic signal through the formation of DISC with FADD and pro-caspase-8 and the activation of a caspase cascade [61]. TRAIL binding with DR5 can also trigger multiple other signaling pathways, including stress kinase activation, conventional NF-κB signaling, and necroptosis [62,63].

**Figure 12:**
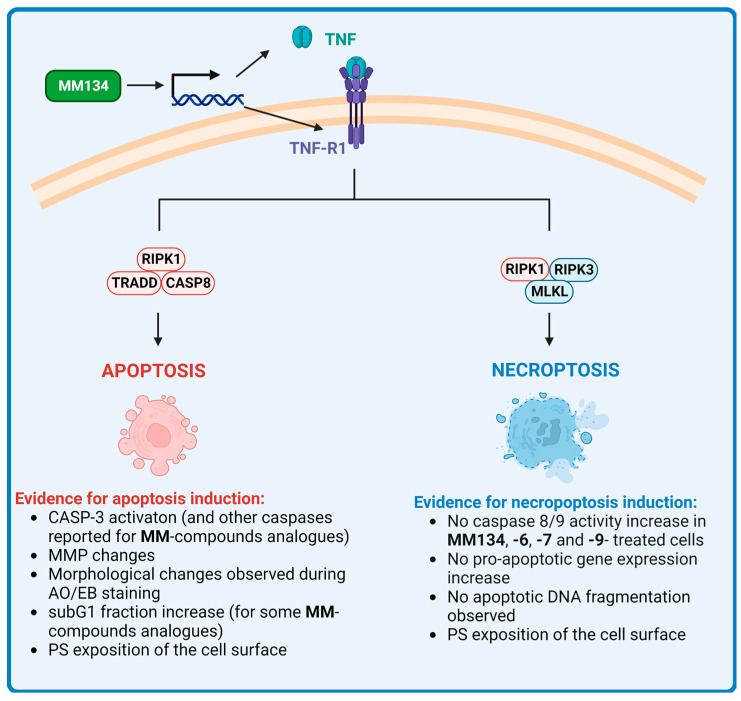
Cell death activated in response to **MM134** (and presumably other pyrazolo[4,3-*e*]tetrazolo[1,5-*b*][1,2,4]triazine sulfonamides (**MM**-compounds)). The treatment of cells with **MM134** induces up-regulation of tumor necrosis factor (TNF) and the tumor necrosis factor receptor superfamily, member 1A (TNFRSF1A; TNF-R1). This event contributes to apoptosis or necroptosis induction with distinct features. Abbreviations: RIPK1/2/3—receptor-interacting serine/threonine kinase 1/2/3; TRADD—tumor necrosis factor receptor type 1-associated DEATH domain; CASP8—caspase-8; MLKL—mixed lineage kinase domain-like pseudokinase.

TNF-receptor I (TNF-R1)-induced apoptosis is also assumed to occur via recruitment of the adaptor FADD and caspase-8 to the receptor complex. While the apoptotic function of TNF-R1 signaling is well-known, it can also increase survival by activating NF-κB. The process by which this choice between cell death and survival is made is ambiguous. TNFR1-induced apoptosis might be caused by two distinct signaling complexes. The first plasma membrane-bound complex (complex I) is made up of TNF-R1, the adaptor protein tumor necrosis factor receptor type 1-associated DEATH domain (TRADD), receptor-interacting serine/threonine kinase 1 (RIPK1), and TNF receptor-associated factor (TRAF2), and activates NF-κB with a pro-survival function. TRADD and RIPK1 then link with caspase-8 to form a cytoplasmic complex (complex II) which contributes to apoptosis. Activation of caspase-8 directs the pathway toward apoptosis, while its suppression leads to necroptosis [64]. Receptor-interacting serine/threonine kinase 1 and 3 (RIPK1 and RIPK3) interact with each other during necroptosis, leading to the development of a functional heterodimer complex that promotes mixed lineage kinase domain-like pseudokinase (MLKL) oligomerization by phosphorylating it. The oligomeric variant of MLKL migrates from the cytosol to the plasma membrane, resulting in the emergence of the pore and inflammatory response [65,66]. During necroptosis, no DNA laddering, caspase activity increases, or pro-apoptotic gene expression occurs. In contrast, similar to apoptotic cells, necroptotic cells may exhibit PS on the outer membrane. Furthermore, as discussed earlier, TNF is the most well-known cytokine triggering the necroptosis pathway [67,68,69]. The above-mentioned findings suggest that the cell death observed following treatment with **MM**-compounds may be a combination of both apoptosis and necroptosis, depending on the cell type, incubation time, and compound concentration (Figure 12).

**Figure 13:**
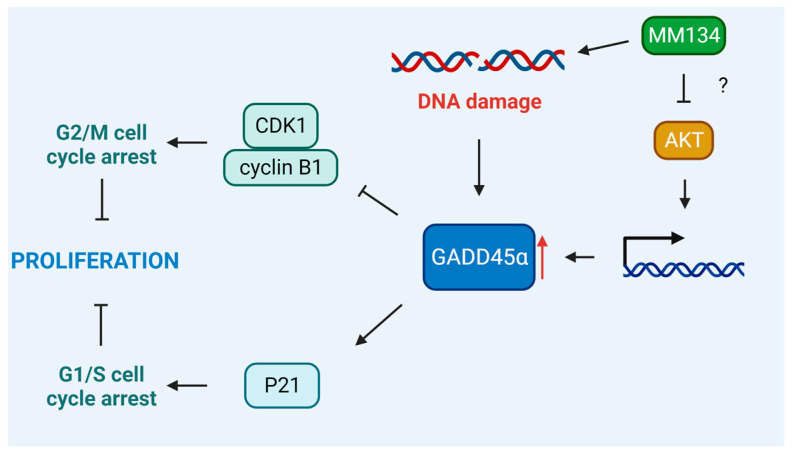
Upregulation of growth arrest and DNA-damage-inducible, alpha (GADD45A) in response to DNA damage induced by **MM**-compounds and inhibition of serine/threonine-protein kinase AKT (as indicated by in silico studies) leads to the activation of cyclin-dependent kinase inhibitor 1 (P21) and disruption of cyclin-dependent kinase 1-cyclin B1 (CDK1-cyclin B1) proteins, contributing to cell cycle proliferation inhibition through G1/S and G2/M phase cell-cycle arrest.

However, the strongest up-regulation of gene expression was observed for GADD45α. GADD45 protein family members undergo rapid activation after DNA damage, leading to cell cycle arrest, DNA repair, and/or cell death. The elevation of GADD45 expression is required for numerous chemotherapeutic agents to mediate their anti-cancer activities, and the absence of GADD45 may negate their effects in cancer cells [70]. GADD45 functions as an upstream effector in the stabilization of cellular tumor antigen p53 (TP53) after DNA damage occurs in cells [71]. The specific DNA-damaging agent appears to be required for GADD45α induction via TP53. In myeloid leukemia cell lines harboring TP53 heterozygous allele, IR can no longer stimulate GADD45α expression, whereas MMS, UV radiation, and serum depletion can still promote GADD45α transcription in breast and colon cancer cell lines with a negative TP53 status [72,73]. Overexpression of GADD45α decreases cell proliferation in a variety of cell types without inducing apoptosis, as evidenced in our investigations [74,75]. This appears to be attributed to the ability of GADD45α to disrupt the interaction between the CDK1/cyclin B1 complex and inhibit its kinase activity, leading to G2/M cell cycle arrest [76,77]. However, through interactions with P21, it can also halt the cell cycle in the G1/S and G2/M phases [70] (Figure 13).

The cell cycle arrest observed by Hermanowicz et al. [12] was shown to be attributed to the upregulation of TP53 expression and downregulation of CDK2. However, we have observed its downregulation in BxPC-3 cells treated with the **MM134** compound. This may indicate a more complex response of cancer cells to **MM**-compounds that might be dependent on GADD45α. In the present work, we also performed extensive in silico studies. In the first stage, we determined the electronic properties of the chemical molecules using HOMO, LUMO, and MESP calculations and re-evaluated the **MM**-compound targets. We found that **MM134**, **-6**, and **-7** compounds form stable complexes with AKT2 kinase, as estimated by the molecular docking, molecular dynamics, and prime MM-GBSA calculations. These complexes and are thought to act through the inhibition of the aforementioned target, while the **MM139** compound could additionally work as a BTK inhibitor. Interestingly, AKT inhibition was found to upregulate the expression of GADD45α independently on TP53 in soft tissue sarcoma cells, indicating the potential mode of activity of the compounds [78] (Figure 13). Nevertheless, enzymatic studies on AKT inhibition should be performed to determine its contribution to the anticancer activity of pyrazole–triazine compounds.

Furthermore, as indicated by the previous investigations, these agents may possess good pharmacokinetic properties and safety profiles, as indicated by the studies of the **MM129** compound. After intraperitoneal treatment, **MM129** exhibited excellent pharmacokinetic features, including fast absorption and a bioavailability of 68.6%. **MM129** also showed a good safety profile in mice, which is supported by the fact that no serious side effects have been reported after in vivo examination [21]. Initially, in silico ADME predictions of **MM134**, **-6**, **-7**, and **-9** suggested that these compounds may exhibit similar or superior properties and should be further investigated for these outcomes.

Altogether, findings of previous studies and the current investigation indicate pyrazolo[4,3-*e*]tetrazolo[1,5-*b*][1,2,4]triazine sulfonamides as an important scaffold for anticancer drug discovery. However, many toxicologic and pharmacodynamic endpoints need to be evaluated in the in vivo setting to confirm the results of these in silico and in vitro studies.

## 4. Materials and Methods

### 4.1. Chemicals

Trypsin-EDTA and all culture media (RPMI-1640, DMEM-F12) were purchased from Biowest (CytoGen, Zgierz, Poland). Phosphate-buffered saline (PBS), 5-Bromo-2′-Deoxyuridine (BrdU), crystal violet, 96% ethanol, fetal bovine serum (FBS), glacial acetic acid, hydrochloric acid (HCl), neutral red (3-amino-7-dimethylamino-2-methyl-phenazine hydrochloride), stabilized penicillin–streptomycin solution, Triton X-100, and Tween 20 were supplied by Merck/Sigma Aldrich Chemical Co. (Burlington, MA, USA). The following chemicals were used for the BrdU incorporation assay: bovine serum albumin (BSA) (Sigma Aldrich Chemical Co. (Burlington, MA, USA)), fluoromount G (Invitrogen, Oxford, UK), normal goat serum (Abcam, Cambridge, UK), paraformaldehyde (PHA) (Polysciences, Inc., Warrington, UK). The Alexa Fluor^®^ 647 mouse anti-BrdU (BD Pharmingen™, San Diego, CA, USA) primary antibody and the Alexa Fluor 594/488 goat anti-mouse (LifeTechnology, Warsaw, Poland) secondary antibody were used. For the caspase activity assays, the Caspase-Glo^®^ 3/7 Assay System (Promega Corporation™, Madison, WI, USA) and Cell Meter™ Caspase 8/9 Activity Apoptosis Assay Kits (BIOKOM, Janki, Poland) were used.

### 4.2. Cell Culture

The BxPC-3 (pancreas adenocarcinoma, ATCC^®^ CRL-1687^TM^) and PC-3 (prostate cancer, ATCC^®^ CRL-1435^TM^) cancer cell lines were obtained from the American Type Culture Collection (ATCC, Rockville, MD, USA). The BxPC-3 cells were grown in RPMI-1640 medium supplemented with 10% (*v*/*v*) fetal bovine serum (FBS) and 1% (*v*/*v*) of both antibiotics (streptomycin and penicillin). The PC-3 cells were grown in DMEM-F12 medium supplemented with 10% (*v*/*v*) fetal bovine serum (FBS) and 1% (*v*/*v*) of both antibiotics (streptomycin and penicillin). The MycoBlue^TM^ Mycoplasma Detector kit (Vazyme Biotech Co., Ltd., Nanjing, China) was used at least every month for the control of mycoplasma contamination in the cell cultures.

The cells were grown at 37 °C in a humidified atmosphere of 5% CO_2_ in the air. The culture medium was changed every 24–48 h. Subculture was performed using 0.25% trypsin/EDTA after the cells reached confluence.

### 4.3. Neutral Red Uptake Assay

The BxPC-3 and PC-3 cells were seeded on 96-well plates at a density of approximately 8–10 × 10^3^ cells per 100 µL medium/well. The cells were allowed to grow for 24 h in controlled conditions (37 °C; 5% CO_2_). Afterward, the cells were subjected to **MM134**, **-6**, **-7**, and **-9** compounds within the range 0.1–3 µM (final concentration of DMSO was <0.5% *v*/*v*) [79] in the culture medium for another 24 h. The experimental design included non-treated controls and blanks (wells without cells). Following 24-h of incubation, the medium containing the compounds was removed, and 100 μL of neutral red (40 µg/mL) in fresh cell culture medium was added to each well of the plate. The cells were incubated in controlled conditions (37 °C, 5% CO_2_) for two hours. The neutral red medium was removed, and the cells were washed with 150 µL of PBS. Afterward, 150 mL of the neutral red destain solution (50% ethanol 96%, 49% deionized water, 1% glacial acetic acid) was added to each well. The plates were shaken on a microtiter plate shaker for 15 min, and the dissolution of neutral red was monitored under a phase-contrast inverted microscope. An absorbance reading was performed at 540 nm using a microplate reader (Power Wave XS BioTek Instruments, Inc., Winooski, VT, USA). The detailed protocol of the neutral red assay was published by other authors [80]. GraphPad Prism 7 software was used to calculate the concentration of **MM**-compound reflecting a 50% inhibition of the uptake (IC_50_). The IC_50_ value was defined as the concentration of the tested compound that led to a reduction of cell pool viability by 50% compared to the negative control (accepted as 100%):

$$\%\;\mathrm{cell}\;\mathrm{viability}=\frac{\left(\mathrm{Absorbance}\;\mathrm{value}\;\mathrm{of}\;\mathrm{treated}\;\mathrm{cells}\;-\mathrm{Absorbance}\;\mathrm{value}\;\mathrm{of}\;\mathrm{blank}\right)}{\left(\mathrm{Absorbance}\;\mathrm{value}\;\mathrm{of}\;\mathrm{untreated}\;\mathrm{cells}-\mathrm{Absorbance}\;\mathrm{value}\;\mathrm{of}\;\mathrm{blank}\right)}\times 100\%$$

### 4.4. Oxidative Stress

BxPC-3 and PC-3 cells were seeded on 96-well black clear bottom microplates at density 12 × 10^5^/mL and cultured in optimal culture conditions (37 °C; 5% CO_2_) for approximately 48 h until they reached the exponential growth phase. Afterward, the culture medium was removed, and the cells were washed three times with 100 µL of PBS. A total of 20 µM of DCFH-DA solution (in PBS) was then added to the cells for 20 min. Afterward, the fluorescent probe was removed, and the cells were washed with PBS and incubated with **MM**-compounds (**MM134**, **-6**, **-7**, and **-9**) in 0.5xIC_50_, IC_50_, and 2xIC_50_ concentrations or H_2_O_2_ at a concentration of 500 µM (positive control). Negative control samples were prepared by incubating the cells in PBS. After the cells were incubated for 1 h at 37 °C in the dark, the ROS induction was assessed. The fluorescence was measured on a SpectraMax i3 Molecular Devices microplate reader using excitation/emission wavelengths of 485 nm and 535 nm.

### 4.5. Caspase 3/7 Detection

A Promega Caspase-Glo^®^ 3/7 Assay System was used for the detection of caspase activity. BxPC-3 and PC-3 cells were seeded at densities of 2 × 10^4^ and 1.5 × 10^4^ per 100 µL well of 96-well white-walled multiwall plates, respectively. After 24 h, the medium was removed, and 100 µL of fresh medium containing **MM**-compounds was added at concentrations of 0.5xIC_50_, IC_50_, and 2xIC_50_ (that were previously estimated in the neutral red uptake assay). Additionally, a positive control (2 µM SN-38) was included in the assay. After 24 h, 100 µL of Caspase-Glo^®^ 3/7 Reagent was added to each well containing 100 µL of blank, negative control cells or cells treated with **MM**-compounds in the culture medium. The plates were placed on a plate shaker and the well contents were mixed (300–500 rpm for 30 s). The plates were incubated at RT for 2 h. Luminescence was recorded using the SpectraMax^®^ i3x Multi-Mode detection platform.

### 4.6. Caspase 8/9 Detection

Cell Meter™ Caspase 8/9 Activity Apoptosis Assay Kits were used for the detection of caspase 8/9 activity in BxPC-3 cells. The cells were seeded at a density of 2 × 10^4^ per 100 µL well of 96-well clear bottom black welled plates. After 24 h, the medium was removed, and 50 µL of fresh medium containing **MM**-compounds at concentrations of 0.5xIC_50_, IC_50_, and 2xIC_50_ (previously obtained in the neural red uptake assay) was added. Additionally, a positive control (2 µM SN-38) was included in the assay. Following 24 h, 50 µL of caspase 8 or 9 substrate working solution was added to each well containing 50 µL of blank, negative control cells or cells treated with **MM**-compounds in the culture medium. The plates were placed on a plate shaker and the well contents were mixed (300–500 rpm for 30 s). The plates were incubated at RT for 1 h. The fluorescence was recorded at Ex/Em = 490/525 nm (Cutoff = 515 nm) using the SpectraMax^®^ i3x Multi-Mode detection platform.

### 4.7. Bromodeoxyuridine (BrdU) Incorporation Assay

PC-3 cells were seeded on coverslips placed on the bottom of 12-well plates at a density of 3 × 10^4^ per 1 mL/well. After 24 h, the medium was removed, and fresh medium (1 mL) containing **MM134**, **-6**, **-7**, and **-9** at concentrations of 0.5xIC_50_, IC_50_, and 2xIC_50_ or 10 µM of cisplatin (used as positive control), was added to each well. After 24 h, the medium with compounds was removed and BrdU was added (final concentration 10 µM/mL). After 24 more hours, the medium was removed, and the cells were washed with 1 mL of PBS. The cells were fixed in 70% cold ethanol and kept at RT for 20 min. Subsequently, the cells were washed twice with PBS (5 min, RT) and once with PBS + 0.5% triton × 100. The cells were incubated with 0.5 mL of PBS and 0.5 mL of 4HCl (30 min, RT). The cells were washed twice with PBS and incubated in 1 mL sodium borate 0.1 M (1 min, RT). The cells were again washed with PBS and incubated with primary anti-BrdU antibody 10 µL/mL in PBS + 1%BSA + 0.5% tween (1 h, RT, dark). The cells were washed with PBS + 0.5% tween twice (2 × 5 min). Thereafter, the cells were incubated with secondary antibody in PBS + 1% BSA + 0.5% tween (1 h). The cells were washed twice with PBS + 0.5% tween (2 × 5 min) and incubated with 4’,6-diamidino-2-phenylindole (DAPI) 1 µg/mL for 15 min, then washed once with PBS (RT, 5 min). The coverslips were mounted using fluoromount G.

The experiment was performed in duplicate, and a total of at least 500 cells per experiment were counted using a fluorescence microscope at 360 nm using CellSens V2.3 (Olympus, Tokyo, Japan) software. The data were presented as % of proliferating cells. The differences between the experimental samples and untreated control or positive control were evaluated using an ANOVA followed by Tukey’s test. A *p*-value less than 0.05 was considered statistically significant (*p* < 0.05).

### 4.8. Clonogenic Assay

BxPC-3 and PC-3 cells were seeded at densities of 100, 200, 500, 1000, and 2000 cells per 3 mL in 6-well plates. The cell culture medium was changed every three days. The colonies were counted ten days after plating the cells to establish plating efficiencies (%).

$$\mathrm{PE}=\frac{\left(\mathrm{Number}\;\mathrm{of}\;\mathrm{counted}\;\mathrm{colonies}\right)}{\left(\mathrm{Number}\;\mathrm{of}\;\mathrm{plated}\;\mathrm{cells}\right)}\times 100\%$$

The cells were seeded at appropriate densities according to the highest plating efficiencies (500 cells/well for BxPC-3 and cells/well for PC-3 cells). The cells were allowed to attach for 2–3 h and were exposed to 0.25xIC_50_, 0.5x IC_50_, and IC_50_ concentrations of **MM**-compounds for 10 days. The medium containing compounds was changed every 2–3 days. After 10 days, the medium containing compounds was removed, and the cells were gently washed with 2 mL of PBS and fixed with 4% formaldehyde for 20 min at RT. The cells were rinsed with PBS, and 0.5% crystal violet in PBS was added for 30 min. The plates were washed by submerging the plates in tap water and air-dried at RT. The colonies were counted using ImageJ V1.53q software. Each treatment was performed in duplicate. The data were analyzed using GraphPad Prism 7.0 software (GraphPad Prism Software Inc., San Diego, CA, USA).

### 4.9. Cell Cycle Analysis

BxPC-3 and PC-3 cells were seeded at a density of 7 × 10^5^ with a 24 h incubation on a 6-well plate in 2 mL of medium. After 24 h incubation in controlled conditions (37 °C; 5% CO_2_), the cells were subjected to **MM134**, **-6**, **-7**, and **-9** compounds in IC_50_ or 0.5 µM concentrations. The experimental design included a vehicle control (final solvent concentration was <0.5% *v/v* DMSO). The cells were incubated for another 24 h (37 °C; 5% CO_2_). Upon exposure, the cells were trypsinized and transferred to cytometric tubes, left for 40 min, and centrifuged at 1400 rpm for 10 min at 4 °C. The supernatant was removed, and the cell pellets were washed with PBS (1 mL). The cells were centrifuged one more time, the supernatant was removed, and cells were re-suspended in 200 µL of ice-cold PBS. The prepared suspensions were transferred to new cytometric tubes containing 1 mL of ice-cold 70% ethanol and mixed. The cells in ethanol were centrifuged (4500 rpm, 10 min, 4 °C), and the supernatant was removed. The cells were incubated with a staining solution (250 µL/tube) containing 5 µL of RNAase A (500 U/mL), 10 µL of PI (500 µg/mL), and 870 µL of PBS for 1 h in the dark at 37 °C and analyzed using flow cytometry.

### 4.10. Apoptotic DNA Fragmentation

BxPC-3 cells were seeded on 12-well plates at a density of 2 × 10^5^. After 24 h, the cells were subjected to **MM134**, **MM136**, **MM137,** and **MM139** compounds in the IC_50_ concentration or 7-Ethyl-10-hydroxycamptothecin (2 µM) (Sigma Aldrich Chemical Co. (Burlington, MA, USA)) for another 24 h. An apoptotic DNA ladder kit (Alexis Biochemical, Lausen, Switzerland) was used to detect nucleosomal DNA fragmentation according to the manufacturer’s instructions. The DNA fragments were visualized on a 1.2% agarose gel.

### 4.11. Gene Expression Analysis

#### 4.11.1. RNA Extraction and cDNA Preparation

BxPC-3 cells were seeded on 25 cm^3^ cell culture flasks Nunc™. After 24 h, the cells were subjected to **MM134** compound used in the IC_50_ concentration or treated with the same volume of DMSO (<0.5% *v*/*v*) for another 24 h. Afterward, an RNA extraction kit (Qiagen, Hilden, Germany) was used to obtain the total RNA. The amount of RNA was measured using a NanoDrop spectrophotometer 2000 (Thermo Scientific, Waltham, MA, USA), and the integrity of the RNA was confirmed using agarose gel electrophoresis. An RT2 HT first strand kit (Qiagen, Hilden, Germany) was used to reverse transcribe up to 1 µg of total RNA to cDNA according to the manufacturer’s instructions. The cDNA was stored at −20 °C for further quantitative PCR (qPCR) analysis.

#### 4.11.2. RT2 Profiler PCR Array Assay

An RT2 Profler PCR Array analysis of human apoptotic gene expression (PAHS-012Z, Qiagen, Hilden, Germany) was performed using a CFX96™ Touch Real-Time PCR Detection System, Bio-Rad, Hercules, CA, USA. PCR Array system using software version 3.5 (Qiagen, Hilden, Germany). The results were used to assess the mRNA expression levels of target genes using the 2^−ΔΔCt^ method.

### 4.12. Computational Analysis

#### 4.12.1. Density Functional Theory (DFT) Calculations

DFT calculations were performed using the Jaguar v11.5 module in Schrödinger to predict the chemical reactivity of the molecules. A hybrid DFT with Berke’s three-parameter exchange potential and Lee–Yang–Parr correlation functional (B3LYP) using basis set 6-31 G++** level was used to optimize the structures. All the DFT calculations were performed in an aqueous environment using PBF. Calculations such as highest occupied molecular orbitals (HOMO), lowest unoccupied molecular orbitals (LUMO), and molecular electrostatic potential (MESP) were performed.

#### 4.12.2. Molecular Docking Studies of MM-Compounds

Triazine sulfonamide analogs were docked into the binding pocket of AKT2 using the LibDock docking engine [31]. The site-feature docking algorithm (LibDock) docks ligands, after removing their hydrogen atoms, into a putative active site guided by binding hotspots. The ligands’ conformations are aligned to polar and apolar receptor interaction sites (i.e., hotspots). LibDock docking follows the following steps: (I) remove hydrogen atoms, (II) rank ligand conformations and prune by solvent accessible surface area (SASA), (III) find hotspots using a grid that is placed into the binding site using polar and apolar probes. The numbers of hotspots are pruned by clustering to a user-defined value. (IV) Dock ligand poses by aligning to binding site hotspots. This is performed by using triplets (i.e., three ligand atoms are aligned to three receptor hotspots). (V) Poses that result in protein clashes are removed, and (VI) a final BFGS pose optimization stage is performed using a simple pair-wise score (similar to piecewise linear potential). The top-scoring ligand poses are retained. Hydrogen atoms are added back to the docked ligands. Optionally, CHARMm minimization can be carried out to reduce steric clashes caused by added hydrogen atoms.

The following LibDock parameters were implemented in the current study: before docking, the Discovery Studio 2.5.5 module CAT-CONFIRM was used to generate a maximum of 255 conformers (not exceeding an energy threshold of 20 kcal/mol from the most stable conformer) for each ligand employing the “best” conformation generation option. A binding site sphere of 8.81 Å radius surrounding the center of the co-crystallized ligand (PDB code: 3O0G or 3GEN) was used to define the binding site. The number of binding site hotspots (polar and a polar) was set to 100. The ligand-to-hotspots matching RMSD tolerance value was set to 0.25 Å. The maximum number of poses saved for each ligand during hotspots matching before final pose minimization was 100. The maximum number of poses to be saved for each ligand in the binding pocket was 100. The minimum LibDock score (poses below this score are not reported) was 100. The maximum number of rigid body minimization steps during the final pose optimization (using the BFGS method) was 50. The maximum number of steric clashes allowed before the pose-hotspot alignment was terminated (specified as a fraction of the heavy atom count) was 0.1. The maximum value for nonpolar solvent accessible surface area for a particular pose to be reported as successful was 15.0 Å^2^. The maximum value for the polar solvent-accessible solvent area for a particular pose to be reported as successful was 5.0 Å^2^. No final ligand minimization was implemented (i.e., in the binding pocket).

#### 4.12.3. Prime MM-GBSA Calculations

The Prime MM-GBSA module of Schrödinger was used to evaluate the binding free energies for the protein–ligand complexes. The protein–ligand complexes were minimized using an optimized potential liquid solvation–all atom (OPLS-AA) force field and generalized Born/Surface (GB/SA) continuum solvent model. The free energy of binding for the protein-ligand complexes was estimated using the following formula:

ΔG_bind_ = G_complex_ − (G_protein_ + G_ligand_)

G = EMM + GSGB + GNP

The energies of the complex, protein, and unbound ligand were represented as G_complex_, G_protein_, and G_ligand_, respectively. The molecular mechanic’s energies (EMM), in addition to the SGB polar solvation model (GSGB) and non-polar solvation (GNP), were together represented as G.

#### 4.12.4. Molecular Dynamic Simulation of MM-Compounds and AKT2

Molecular dynamics (MD) simulations were performed using Gromacs 2020.4 version [81] to examine the stability of protein–ligand complexes obtained from molecular docking studies. The necessary input files were prepared via the CHARMM-GUI server [82]. Topology files of proteins and compounds were prepared using AMBER99SB force fields [83]. Protein–ligand complex structures were solvated using the TIP3 water model, using the rectangular box type, 10 Å away from the protein–ligand complexes, and neutralized by adding 0.15 KCl salt. The created system was minimized to 5000 nsteps with the steep integrator. The system was equilibrated using the Nose–Hoover [84,85,86] and Parrinello–Rahman algorithms [87] with 0.3 ns duration NVT/NPT ensemble steps at 300 K and 1 atm pressure. A 200-ns MD simulation was run at 2 fs, and 2000 frames were recorded. The root mean square deviation (RMSD), the root mean square fluctuation (RMSF), hydrogen bonds between protein and ligand, and radius of gyration (Rg) were measured using gmx scripts. The binding free energy molecular mechanics Poisson–Boltzmann surface area (MM-PBSA) was calculated using 100 frames between 150 ns and 200 ns with the g_mmpbsa tool (V1.6) [88]. RMSD, RMSF, H bonds, and Rg plots were created using QtGrace v2.0.6. MD trajectory videos were created using PyMol Molecular Graphics System version 2.5.2 software, and protein–ligand binding pose visualizations were created using BIOVIA Discovery Studio Visualizer v21.

## Figures and Tables

**Table 1:** Differentially expressed genes (*p* < 0.05) between **MM134**-treated cells and control (DMSO-treated cells). Upregulated genes str marked in red, while down-regulated str indicated in green.

Gene Symbol	Description	Fold Regulation	*p*-Value
**BCL10**	B-cell CLL/lymphoma 10	2.50	0.019800
**GADD45A**	Growth arrest and DNA-damage-inducible, alpha	6.24	0.000393
**RIPK2**	Receptor-interacting serine-threonine kinase 2	2.83	0.002422
**TNF**	Tumor necrosis factor	2.96	0.042660
**TNFRSF10B**	Tumor necrosis factor receptor superfamily, member 10b	4.36	0.001260
**TNFRSF1A**	Tumor necrosis factor receptor superfamily, member 1A	2.18	0.018576
**ABL1**	C-abl oncogene 1, non-receptor tyrosine kinase	−2.65	0.006966
**AIFM1**	Apoptosis-inducing factor, mitochondrion-associated, 1	−2.48	0.001081
**APAF1**	Apoptotic peptidase activating factor 1	−2.74	0.002161
**BAD**	BCL2-associated agonist of cell death	−2.36	0.006358
**BAG1**	BCL2-associated athanogene	−2.11	0.006014
**BCL2L10**	BCL2-like 10 (apoptosis facilitator)	−2.38	0.019800
**BIRC5**	Baculoviral IAP repeat containing 5	−5.01	0.003025
**BNIP3**	BCL2/adenovirus E1B 19kDa interacting protein 3	−2.04	0.009433
**BNIP3L**	BCL2/adenovirus E1B 19kDa interacting protein 3-like	−2.01	0.005716
**CASP1**	Caspase 1, apoptosis-related cysteine peptidase (interleukin 1, beta, convertase)	−3.76	0.033341
**CASP14**	Caspase 14, apoptosis-related cysteine peptidase	−2.83	0.000610
**CASP2**	Caspase 2, apoptosis-related cysteine peptidase	−2.63	0.009949
**CASP6**	Caspase 6, apoptosis-related cysteine peptidase	−2.87	0.016235
**CD27**	CD27 molecule	−3.05	0.001928
**CD70**	CD70 molecule	−3.67	0.000879
**CIDEA**	Cell death-inducing DFFA-like effector a	−2.03	0.001025
**CIDEB**	Cell death-inducing DFFA-like effector b	−2.15	0.002795
**CRADD**	CASP2 and RIPK1 domain containing adaptor with death domain	−3.37	0.001127
**DFFA**	DNA fragmentation factor, 45kDa, alpha polypeptide	−2.34	0.020318
**FADD**	Fas (TNFRSF6)-associated via death domain	−3.68	0.000243
**FAS**	Fas (TNF receptor superfamily, member 6)	−5.41	0.000181
**NAIP**	NLR family, apoptosis inhibitory protein	−6.35	0.025237
**NOD1**	Nucleotide-binding oligomerization domain containing 1	−3.13	0.000604
**PYCARD**	PYD and CARD domain containing	−2.41	0.010219
**TNFRSF11B**	Tumor necrosis factor receptor superfamily, member 11b	−3.85	0.000160
**TNFSF10**	Tumor necrosis factor (ligand) superfamily, member 10	−6.25	0.000372
**TP53**	Tumor protein p53	−3.84	0.023076

**Table 2:** Density functional theory calculation of the **MM134**, **MM136**, **MM137**, and **MM139** compounds.

Compound ID	HOMO (eV)	LUMO (eV)	HLG (eV)
**MM134**	−0.238	−0.224	−0.014
**MM136**	−0.239	−0.208	−0.030
**MM137**	−0.243	−0.220	−0.022
**MM139**	−0.250	−0.201	−0.049

**Table 3:** Libdock-based docking conditions for **MM134**, **-6**, **-7**, and **-9** compounds with cancer targets and their corresponding binding energies.

Drug Target (PDB Code)	Docking Conditions		Binding Energy (BE) ^b^ of Inhibitors			
	Explicit Water ^a^	Scoring Function	MM134	MM136	MM137	MM139
AKT2 (3D0E)	Absent	Ligscore1	−70.158	−96.359	−52.722	−15.722
BTK (3GEN)	Present	Ligscore2	−7.85	−15.726	−32.198	−48.185
CHK1 (2YM8)	Present	Ligscore2	−31.609	−10.281	−22.055	−9.749
PD-L1 (7BEA)	Absent	Ligscore2	−60.955	−8.811	−24.089	−4.587

^a^: Crystallographic explicit water of hydration. ^b^: Unit of binding energy kcal/mol.

**Table 5:** MM-PBSA calculations of binding free energy of **MM134**, **MM136**, **MM137**, **MM139** and cocrystal ligand G39 with AKT2.

Parameters (Energy)	AKT2–MM134 (kJ/mol)	AKT2–MM136 (kJ/mol)	AKT2–MM137 (kJ/mol)	AKT2–MM139 (kJ/mol)	AKT2–G39 (kJ/mol)
Van der Waals	226.8 ± 16.3	−198.8 ± 11.3	−145.9 ± 16.4	−105.2 ± 19.4	−221.7 ± 15.5
Electrostatic	−58.3 ± 15.5	−11.6 ± 10.8	−43.6 ± 19.1	−29.3 ± 17.1	−86.1 ± 16.2
Polar solvation	221.5 ± 38.2	96.9 ± 22.8	155.9 ± 33.2	70.7 ± 27.7	181.5 ± 32.0
SASA	−21.1 ± 1.1	−16.6 ± 0.9	−15.6 ± 1.6	−10.9 ± 2.3	−20.5 ± 1.1
Binding free	−84.7 ± 23.5	−130.1 ± 20.6	−49.2 ± 25.9	−74.7 ± 16.6	−146.9 ± 38.9

## Data Availability

The data presented in this study are available in the main text of this article/Supplementary Materials of this article or on request from the corresponding author.

## Supplementary Materials

The supporting information can be downloaded at: https://www.mdpi.com/article/10.3390/ijms241310959/s1.

## Abbreviations

ABL	tyrosine-protein kinase ABL
ADMET	absorption, distribution, metabolism, excretion, and toxicity
AIFM1	apoptosis-inducing factor, mitochondrion-associated, 1
AKT	serine/threonine-protein kinase AKT
AO/EB	acridine orange/ethidium bromide
APAF-1	apoptotic protease-activating factor 1
BAD	BCL2-associated agonist of cell death
BAG1	BCL2-associated athanogene
BCL10	B-cell CLL/lymphoma 10
BCL2L10	BCL2-like 10 (apoptosis facilitator)
BE	binding energy
BIRC5	baculoviral IAP repeat containing 5
BNIP3	BCL2/adenovirus E1B 19 kDa interacting protein 3
BNIP3L	BCL2/adenovirus E1B 19 kDa interacting protein 3-like
BrdU	Bromodeoxyuridine
BSA	bovine serum albumin
BTK	Bruton’s tyrosine kinase
CAs	carbonic anhydrases
CASP1	caspase 1, apoptosis-related cysteine peptidase (interleukin 1, beta, convertase)
CASP14	caspase 14, apoptosis-related cysteine peptidase
CASP2	caspase 2, apoptosis-related cysteine peptidase
CASP6	caspase 6, apoptosis-related cysteine peptidase
CARD	caspase recruitment domain
CDKs	cyclin-dependent kinases
CD27	CD27 molecule
CD70	CD70 molecule
CHK1	serine/threonine-protein kinase CHK1
CIDEA/B	Cell death-inducing DFFA-like effector A/B
CLL	chronic lymphocytic leukemia
CRADD	CASP2 and RIPK1 domain containing adaptor with death domain
CYP	cytochrome
DCF	2′,7′-dichlorofluorescein
DCFH2	2′,7′-dichlorodihydrofluorescein
DCFH-DA	2,7-dichlorodihydrofluorescein diacetate
DFFA	DNA fragmentation factor, 45 kDa, alpha polypeptide
DISC	death-inducing signaling complex
DPT	developmental toxicity potential
EMM	molecular mechanic’s energies
ESP	electrostatic potential
FADD	FAS-associated death domain protein
FAS	Fas (TNF receptor superfamily, member 6)
FBS	fetal bovine serum
GADD45α	growth arrest and DNA-damage-inducible, alpha
GB/SA	generalized Born/Surface continuum solvent model
GNP	non-polar solvation
HCl	hydrochloric acid
HLG	HOMO-LUMO energy gap
HOMO	highest occupied molecular orbital
LD50	50% lethal dose
LOAEL	lowest observable adverse effects level
LUMO	lowest occupied molecular orbital
MCL	mantle cell lymphoma
MD	molecular dynamics
MESP	molecular electrostatic potential
MLKL	mixed lineage kinase domain like pseudokinase
MMP	mitochondrial membrane potential
mTOR	mammalian target of rapamycin kinase
NAIP	NLR family, apoptosis inhibitory protein
NF-κB	nuclear factor kappa-B
NOD1	nucleotide-binding oligomerization domain containing 1
OPLS-AA	optimized potential liquid solvation-all atom
OPS	optimal prediction space
PBS	phosphate-buffered saline
P21	Cyclin-dependent kinase inhibitor 1 (also known as Waf1/Cip1)
PD-1	programmed cell death protein 1
PD-L1	programmed death-ligand 1
PHA	Paraformaldehyde
PI	propidium iodide
PPB	plasma protein binding
PS	phosphatidylserine
PYCARD	PYD and CARD domain containing
QSAR	quantitative structure-activity relationship
RFU	relative fluorescence units
RIPK2	receptor-interacting serine-threonine kinase 2
RMSD	trajectory root mean square deviation
RMSF	root mean square fluctuation
ROS	reactive oxygen species
SASA	solvent accessible surface area
sICAM-1	soluble intercellular adhesion molecule-1
SMILES	simplified molecular input line entry system
TOPKAT	toxicity prediction by computer-aided technology
TNF	tumor necrosis factor
TNFRSF10B	tumor necrosis factor receptor superfamily, member 10b
TNFRSF11B	tumor necrosis factor receptor superfamily, member 11b
TNFRSF1A	tumor necrosis factor receptor superfamily, member 1A
TNFSF10	tumor necrosis factor (ligand) superfamily, member 10
TP53	cellular tumor antigen p53
TRADD	tumor necrosis factor receptor type 1-associated DEATH domain
TRAF2	TNF receptor-associated factor 2
TRAIL	tumor necrosis factor-related apoptosis inducing ligand
λem	emission wavelength
λex	excitation wavelength
