# Supplementary material for: Pyrazolo[4,3-e]tetrazolo[1,5-b][1,2,4]triazine Sulfonamides as an Important Scaffold for Anticancer Drug Discovery—In Vitro and In Silico Evaluation
Source: Int J Mol Sci. 2023 Jun 30;24(13):10959. doi: 10.3390/ijms241310959 (PMC10342037; doi:10.3390/ijms241310959)
Supplement: Supplementary file 1 [file ijms-24-10959-s001.zip › ijms-2483917-supplementary.pdf]

International Journal of *Molecular Sciences*

MDPI

Supplementary Information

Pyrazolo[4,3-*e*]tetrazolo[1,5-*b*][1,2,4]triazine Sulfonamides as an Important Scaffold for Anticancer Drug Discovery—In Vitro and In Silico Evaluation

Mateusz Kciuk [1,2], Beata Marciniak [1], Ismail Celik [3], Enfale Zerroug [4], Amit Dubey [5,6], Rajamanikandan Sundaraj [7], Somdutt Mujwar [8], Karol Bukowski [1], Mariusz Mojzych [9] and Renata Kontek [1,*]

1 Department of Molecular Biotechnology and Genetics, University of Lodz, Banacha 12/16, 90-237 Lodz, Poland; mateusz.kciuk@edu.uni.lodz.pl (M.K.); beata.marciniak@biol.uni.lodz.pl (B.M.); karol.bukowski@edu.uni.lodz.pl (K.B.)
2 Doctoral School of Exact and Natural Sciences, University of Lodz, Banacha Street 12/16, 90-237 Lodz, Poland
3 Department of Pharmaceutical Chemistry, Faculty of Pharmacy, Erciyes University, Kayseri 38280, Turkey; ismailcelik@erciyes.edu.tr
4 Group of Computational and Pharmaceutical Chemistry, LMCE Laboratory, University of Biskra, BP 145, Biskra 07000, Algeria; anfelzerroug220@gmail.com
5 Computational Chemistry and Drug Discovery Division, Quanta Calculus, Greater Noida 274203, Uttar Prades, India; ameetbioinfo@gmail.com
6 Department of Pharmacology, Saveetha Dental College and Hospital, Saveetha Institute of Medical and Technical Sciences, Chennai 602105, Tamil Nadu, India
7 Centre for Drug Discovery, Department of Biochemistry, Karpagam Academy of Higher Education, Coimbatore 641021, Tamil Nadu, India; mani.bioinfor@gmail.com
8 Chitkara College of Pharmacy, Chitkara University, Rajpura 140401, Punjab, India; somduttmujwar@gmail.com
9 Department of Chemistry, Siedlce University of Natural Sciences and Humanities, 3 Maja 54, 08-110 Siedlce, Poland; mariusz.mojzych@uph.edu.pl
* Correspondence: renata.kontek@biol.uni.lodz.pl

Table S1. 24-h IC_{50} values [μM] obtained from two independent experiments (1,2) with corresponding coefficients of determination (R^2) and calculated mean IC_{50} values ± SD.

MM-compounds	$IC_{50\ (1)}$	$R^2_{(1)}$	$IC_{50\ (2)}$	$R^2_{(2)}$	Mean IC_{50} value ± SD [μM]
			**BxPC-3 cells**		
**MM134**	**0.36**	0.93	**0.35**	0.97	**0.35 ± 0.007**
**MM136**	**0.24**	0.99	**0.3**	0.99	**0.27 ± 0.04**
**MM137**	**0.17**	0.95	**0.19**	0.94	**0.18 ± 0.014**
**MM139**	**0.27**	0.99	**0.3**	0.98	**0.28 ± 0.02**
			**PC-3 cells**		
**MM134**	**0.07**	0.89	**0.13**	0.97	**0.1 ± 0.04**
**MM136**	**0.12**	0.99	**0.14**	0.98	**0.13 ± 0.014**
**MM137**	**0.06**	0.99	**0.06**	0.99	**0.06**
**MM139**	**0.21**	0.99	**0.14**	0.99	**0.17 ± 0.05**

Table S2. Effect of 24-h incubation of PC-3 cells with IC_{50} and $2xIC_{50}$ concentrations of **MM**-compounds.

Compound	Concentration	% of proliferating cells
**MM134**	IC_{50}	72.6 ± 1.48
	$2xIC_{50}$	62.2 ± 4.45
**MM136**	IC_{50}	68.4 ± 1.27
	$2xIC_{50}$	56.7 ± 10.1
**MM137**	$IC_{50}/2xIC_{50}$	65.6 ± 5.79
**MM139**	IC_{50}	68 ± 12.7
	$2xIC_{50}$	46.8 ± 0.78

Table S3. MTT assay results of **MM**-compounds described in the literature and obtained from the non-published source. Cytotoxic/cytostatic effects of compounds were described for multiple cancer cell lines: BxPC-3 (pancreas adenocarcinoma), PC-3 (prostate cancer), cervical cancer (HeLa), colorectal adenocarcinoma (DLD1/HCT116/HT29) and normal cells: human lung fibroblasts (WI-38) and human peripheral blood mononuclear cells (PBMCs). The results were obtained for the 72-h incubation of the compounds with cells (or 24-h as indicated by pound sign; #). Yellow marking – cell lines investigated in this manuscript, green marking – normal cell lines.

**Compound**	**Cell line**							
	**IC_{50} value**							
	**BxPC-3**	**PC-3**	**HeLa**	**DLD-1**	**HCT116**	**HT-29**	**WI-28**	**PBMCs**
MM129	0.26	0.36	0.9	3.1#	0.6	3.1#	N/D	1.11
MM130	0.17	0.22	0.59	N/D	0.44	N/D	N/D	0.77
MM131	0.13	0.17	0.41	3.4#	0.39	3.9#	N/D	0.62
MM134	0.32	0.16	N/D	0.27*	0.38	0.2*	0.65	N/D
MM136	0.25	0.13	N/D	0.13*	0.25	0.12*	0.48	N/D
MM137	0.16	0.11	N/D	0.08*	0.14	0.08*	0.27	N/D
MM139	0.33	0.17	N/D	0.12*	0.35	0.09*	0.64	N/D

N/D – non determined; * - non published data; #- 24-h incubation.

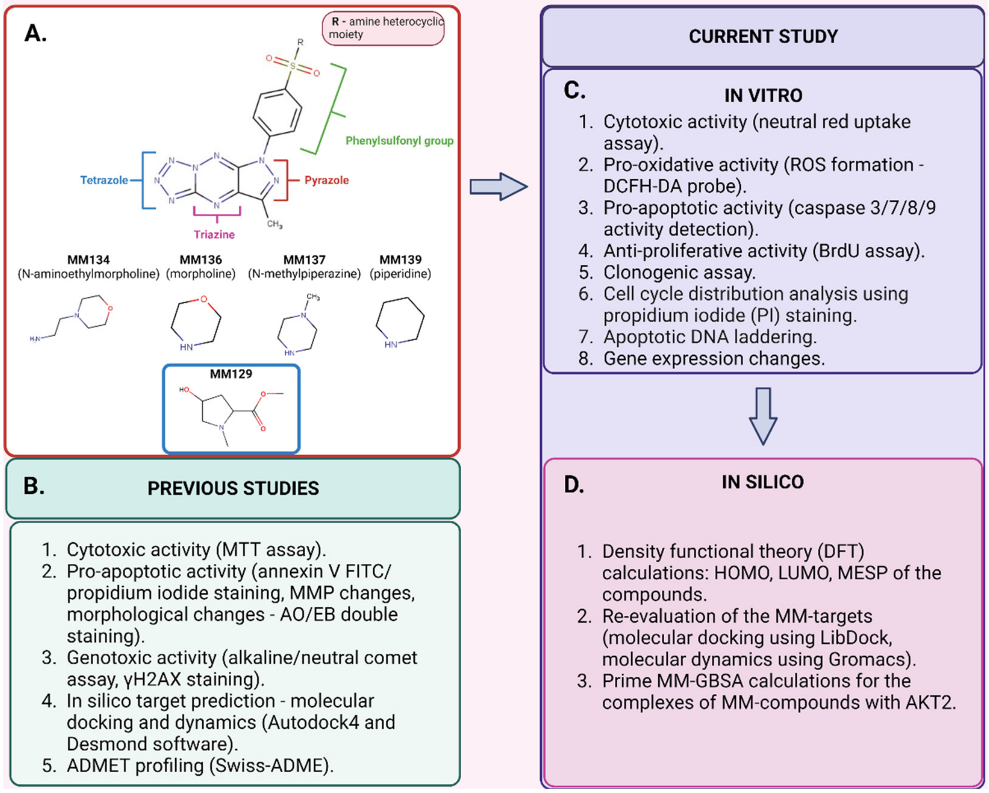

Figure S1. The study workflow. A) Four pyrazolo[4,3-*e*]tetrazolo[1,5-*b*][1,2,4]triazine sulphonamides (**MM**-compounds) were investigated in the current study. These compounds differ in the amine heterocyclic moiety which constitutes N-aminoethylmorpholine (**MM134**), morpholine (**MM136**), N-methylpiperazine (**MM137**), and piperidine (**MM139**). The compounds are close analogues of methyl 4-hydroxy-1-[4-(7-methyl-5H-pyrazolo[4,3-*e*]tetrazolo[1,5-*b*][1,2,4]triazin-5-yl)phenylsulfo-nyl]pyrrolidine-2-carboxylate (**MM129)** compound which efficiently limits cell viability via inhibition of Bruton's tyrosine kinase (BTK) [10] and exhibits antitumor activity in colon cancer xenograft mice as a result of a decrease in serine/threonine-protein kinase AKT (AKT), cyclin-dependent kinase 2 (CDK2), mammalian target of rapamycin kinase (mTOR), and programmed death-ligand 1 (PD-L1) expression [18]. B) In the previous studies [11] **MM34**, **-6**, **-7** and **-9** compounds used in micromolar concentrations showed cytotoxic, genotoxic, and pro-apoptotic properties. Additionally, compounds exhibited favorable ADMET properties and were predicted to act through the AKT-mTOR pathway or BTK and CHK1 kinase inhibition and were indicated to disrupt the PD-1/PD-L1 interaction in the in silico investigation. C) In the current study, cytotoxic and pro-apoptotic properties of the compounds were investigated, together with their anti-clonogenic, anti-proliferative, and pro-oxidative properties. Furthermore, we explored gene expression changes in BxPC-3 cells following treatment with the most pro-apoptotic **MM134** compound D) The in vitro studies were complemented with detailed in silico investigation including the re-evaluation of the interaction between the compounds and predicted targets to select the most promising hits for further investigations. The compounds' properties were investigated with HOMO, LUMO and MESP calculations. Abbreviations: ADMET—absorption, distribution, metabolism, excretion, and toxicity; AKT—serine/threonine-protein kinase AKT; BTK—Bruton's tyrosine kinase; CHK1—serine/threonine-protein kinase CHK1; HOMO—highest occupied molecular orbital; MESP— molecular electrostatic potential; mTOR—mammalian target of rapamycin kinase; LUMO—lowest occupied molecular orbital; PD-1—programmed cell death protein 1; PD-L1—programmed death-ligand 1. Created with BioRender.com accessed on 24 April 2023.

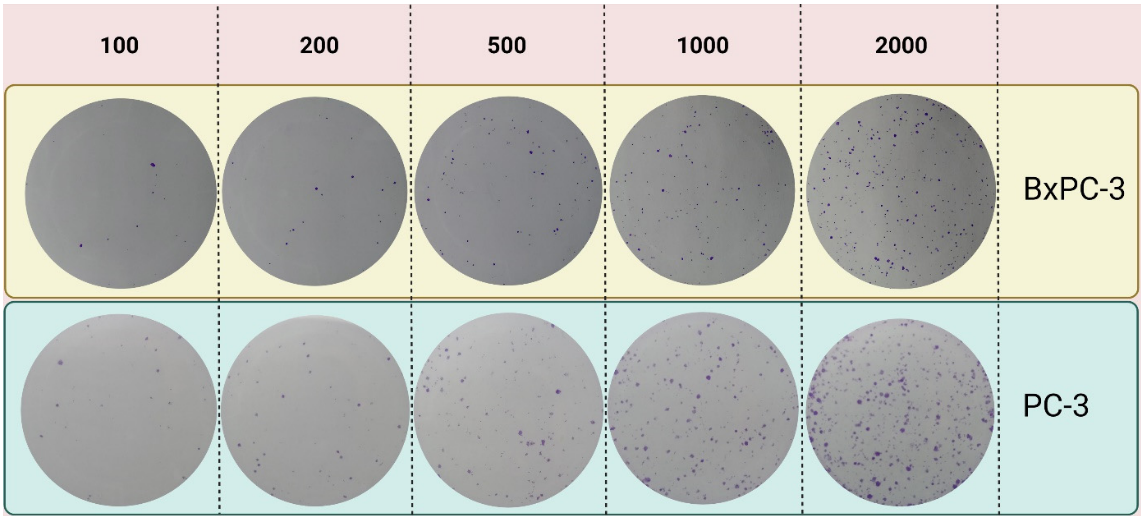

Figure S2. Examples of images obtained after seeding 100, 200, 500, 1000, and 2000 of BxPC-3 or PC-3 cells. Created with BioRender.com accessed on 24 April 2023.

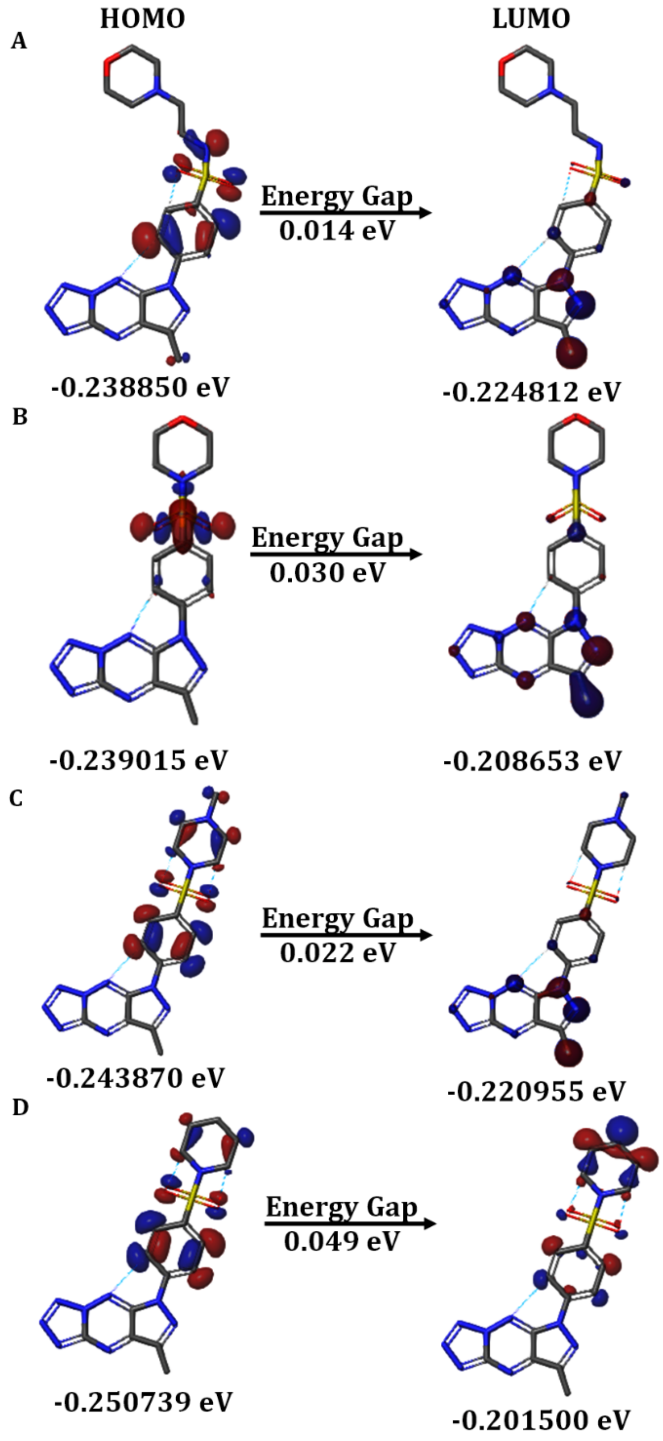

Figure S3. HOMO and LUMO distribution profile of the compounds. A) **MM134**, B) **MM136**, C) **MM137**, and D) **MM139**.

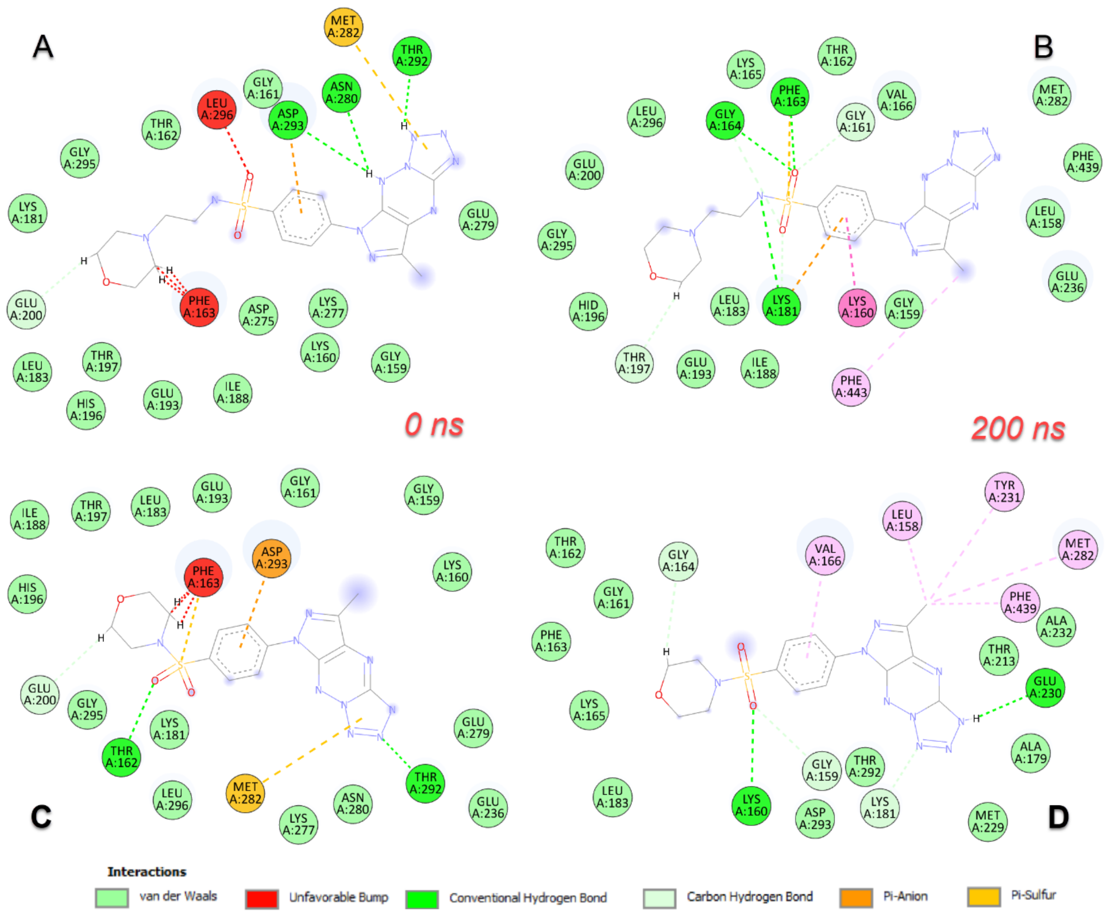

Figure S4. Diagrams of protein-ligand interactions of **AKT2** & **MM134** and **AKT2** & **MM136** complexes at the middle and end of 200 ns time simulation. (**A**) 0 ns interactions in **AKT2** & **MM134** complex, (**B**) 2D interactions at 200 ns, and (**C**) Schematic protein-ligand interaction diagrams of the **AKT2** & **MM136** complex at 0 ns and (**D**) at 200 ns of MD simulation.

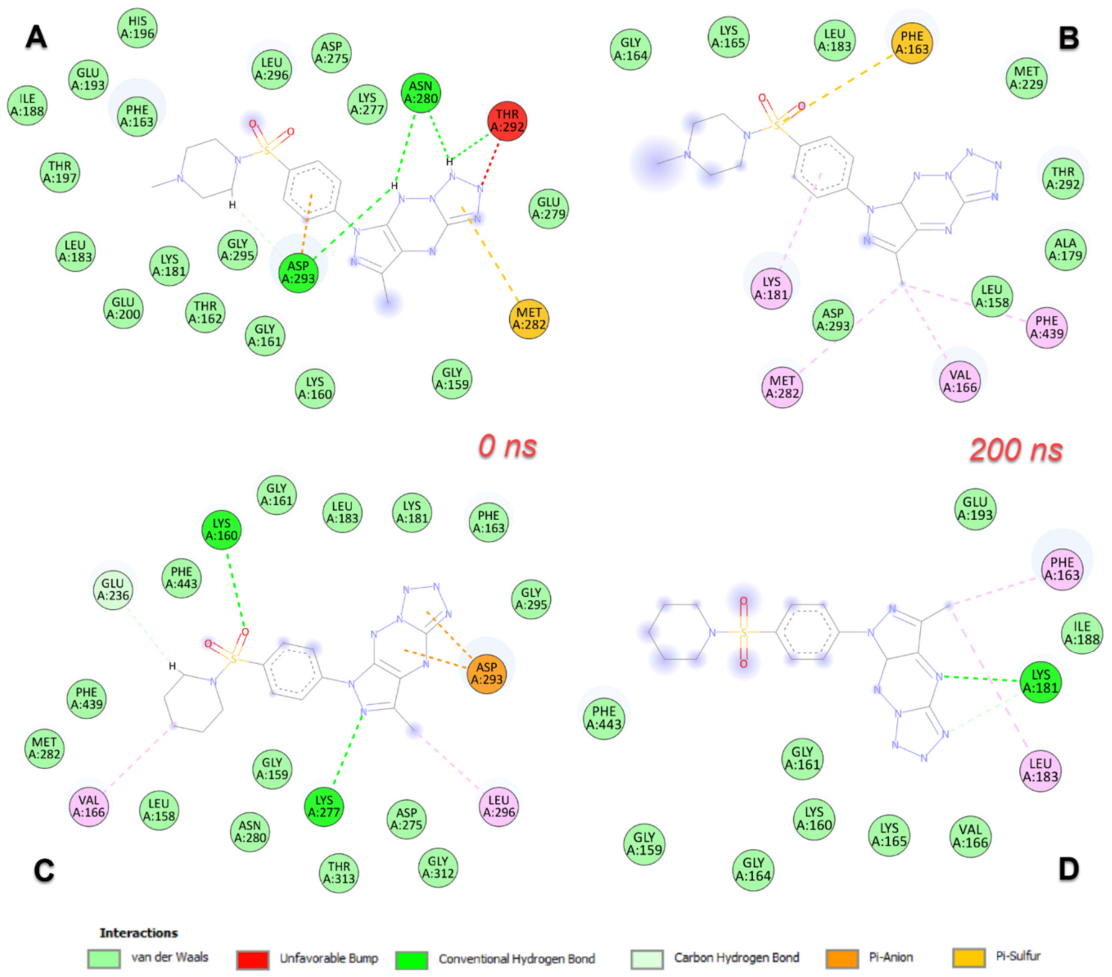

Figure S5. Diagrams of protein-ligand interactions of AKT2 & **MM137** and AKT2 & **MM139** complexes at the middle and end of 200 ns time simulation. (**A**) 0 ns interactions in AKT2 & **MM137** complex, (**B**) 2D interactions at 200 ns, and (**C**) Schematic protein-ligand interaction diagrams of the AKT2 & **MM139** complex at 0 ns and (**D**) at 200 ns of MD simulation.

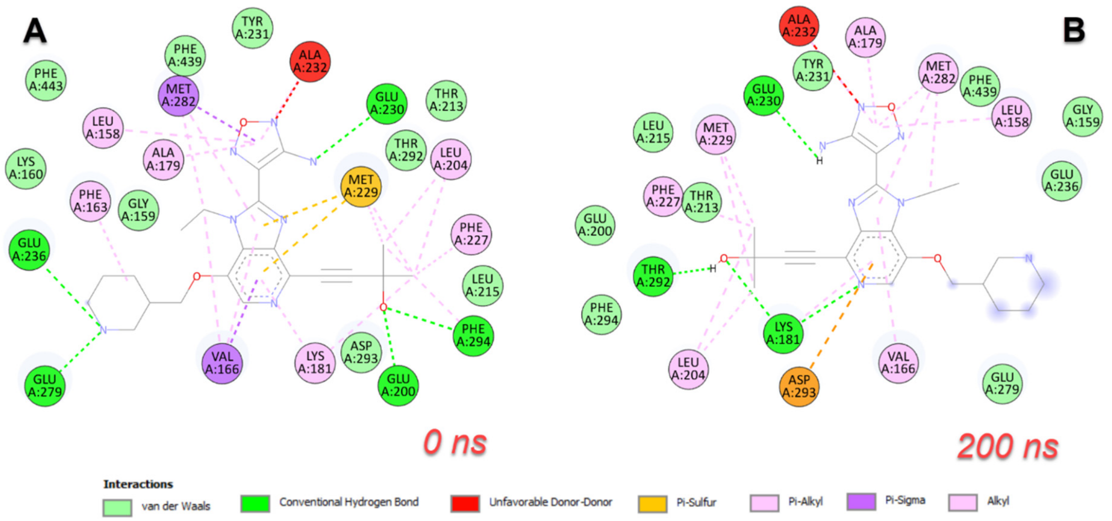

Figure S6. Diagrams of protein-ligand interactions of AKT2 & G39 complexes at the 0 ns and end of 200 ns time simulation. (**A**) 0 ns interactions in AKT2 & G39 complex, (**B**) 2D interactions at 200 ns, and of MD simulation.

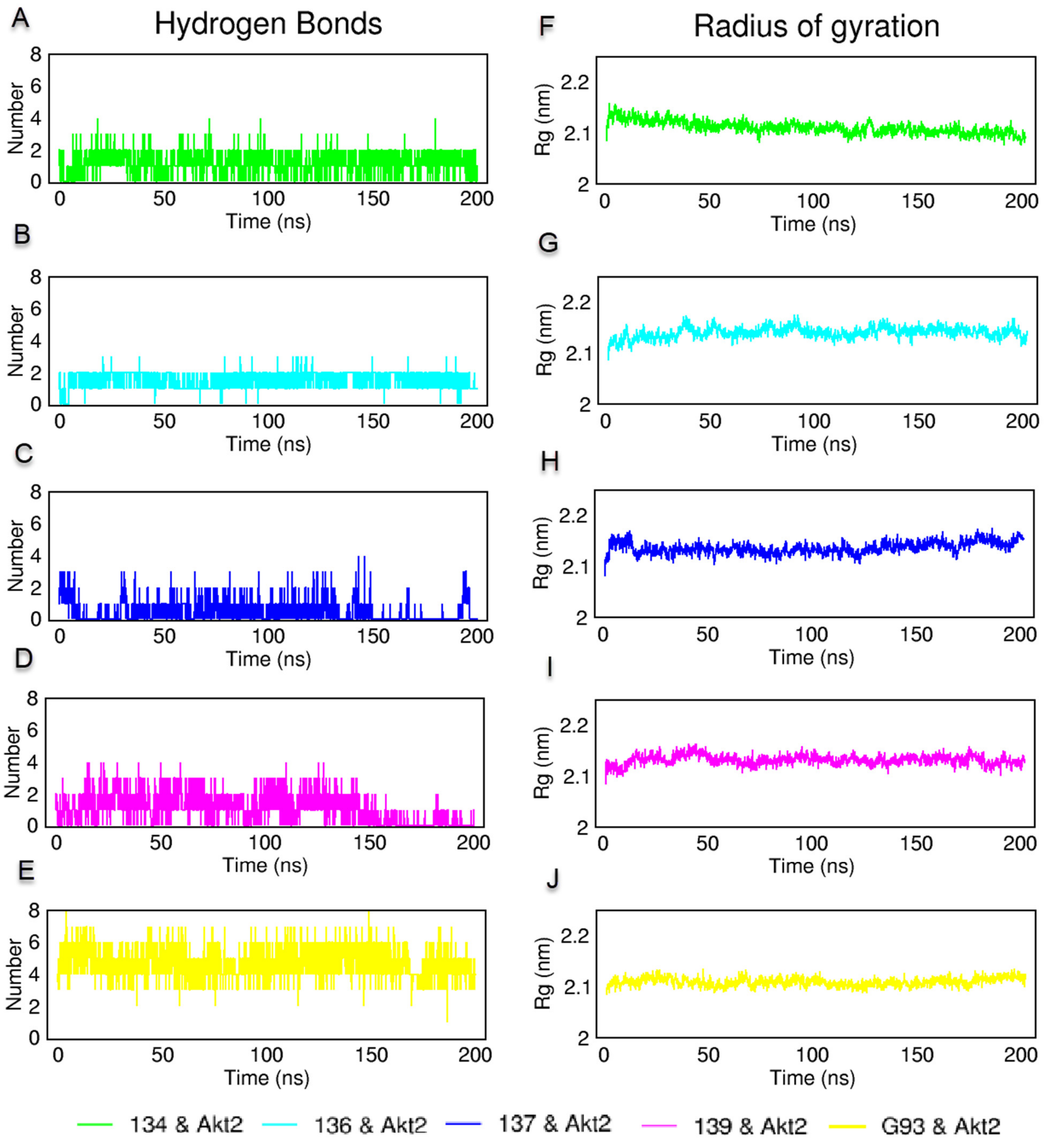

Figure S7. Hydrogen bonding and compactness analysis from molecular dynamics simulation: (A-E) H bond number and exchange between **MM134** & AKT2, **MM136** & AKT2, **MM137** & AKT2, **MM139** & AKT2, and G39 & AKT2, and (F-J) radius of gyration (Rg) measurement of these protein-ligand complexes for 200 ns.

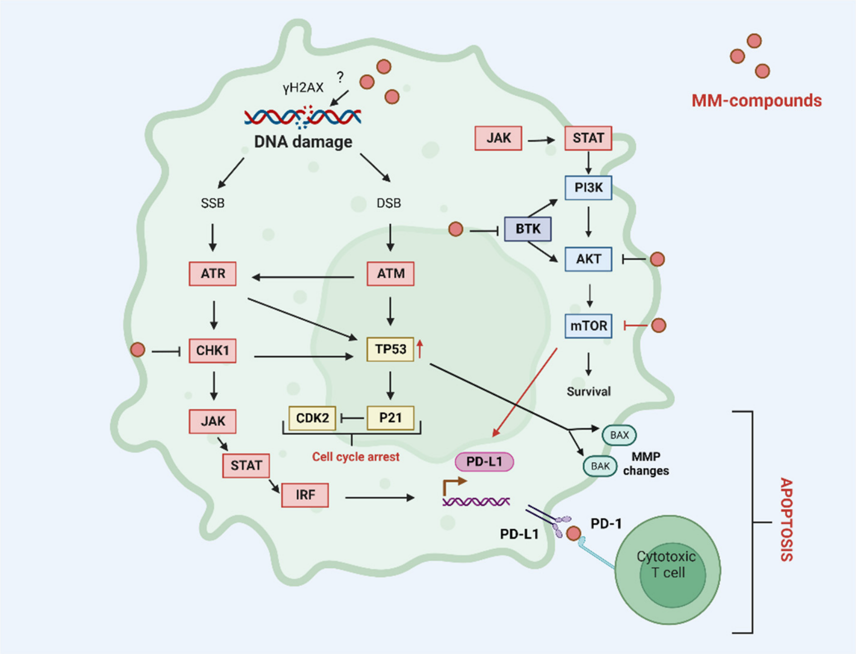

Figure S8. Possible molecular mechanism of biological activity of the **MM**-compounds. Adapted from [8] and modified. Based on [7,10,18,38,81]. Created with BioRender.com accessed on 24 April 2023.

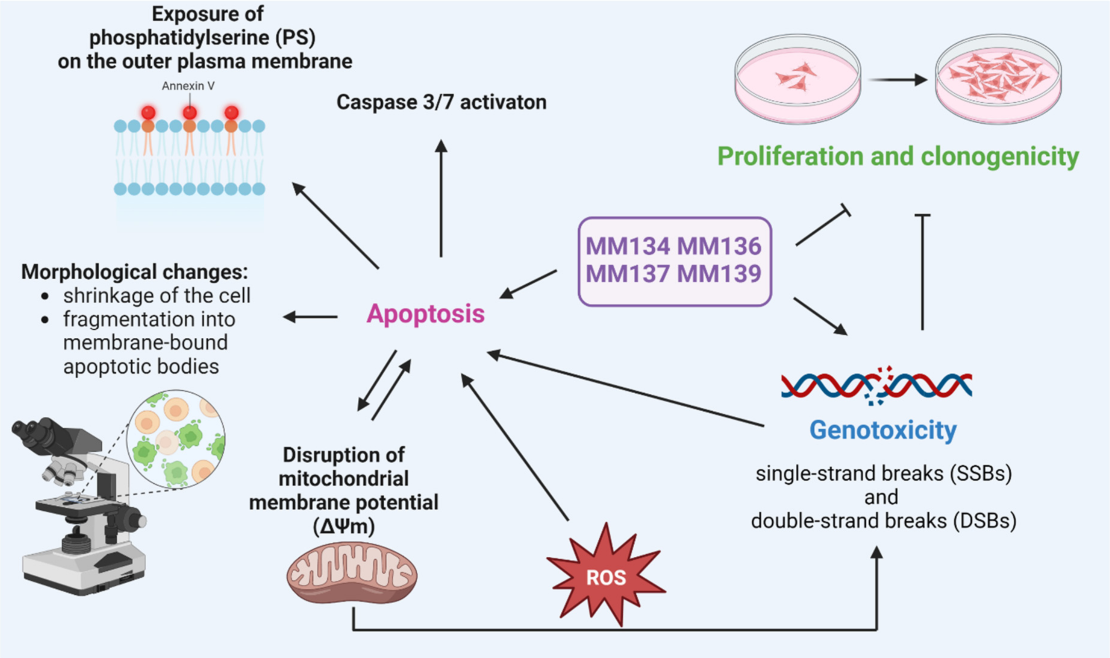

Figure S9. Summary of biological activity of **MM134**, **-6**, **-7,** and **-9** compounds reported in the literature. Based on [8,11]. Created with BioRender.com accessed on 24 April 2023.
